# Effectiveness of current and future regimens for treating genotype 3 hepatitis C virus infection: a large-scale systematic review

**DOI:** 10.1186/s12879-017-2820-z

**Published:** 2017-11-16

**Authors:** Hosnieh Fathi, Andrew Clark, Nathan R. Hill, Geoffrey Dusheiko

**Affiliations:** 1 Almirall Ltd, London, UB11 1BT UK; 2 Bristol-Myers Squibb Pharmaceuticals Ltd, London, UB8 1DH UK; 3 UCL Medical School, Kings College Hospital, Denmark Hill, London, SE5 9RS UK

**Keywords:** Hepatitis C virus, Genotype 3, Direct-acting antiviral, Cirrhosis, Co-infection, Systematic literature review

## Abstract

**Background:**

Six distinct genetic variants (genotypes 1 − 6) of hepatitis C virus (HCV) exist globally. Certain genotypes are more prevalent in particular countries or regions than in others but, globally, genotype 3 (GT3) is the second most common. Patients infected with HCV GT1, 2, 4, 5 or 6 recover to a greater extent, as measured by sustained virological response (SVR), following treatment with regimens based on direct-acting antivirals (DAAs) than after treatment with older regimens based on pegylated interferon (Peg-IFN). GT3, however, is regarded as being more difficult to treat as it is a relatively aggressive genotype, associated with greater liver damage and cancer risk; some subgroups of patients with GT3 infection are less responsive to current licensed DAA treatments. Newer DAAs have become available or are in development.

**Methods:**

According to PRISMA guidance, we conducted a systematic review (and descriptive statistical analysis) of data in the public domain from relevant clinical trial or observational (real-world) study publications within a 5-year period (February 2011 to May 2016) identified by PubMed, Medline In-Process, and Embase searches. This was supplemented with a search of five non-indexed literature sources, comprising annual conferences of the AASLD, APASL, CROI, EASL, and WHO, restricted to a 1-year period (April 2015 to May 2016).

**Results:**

Of the all-oral regimens, the efficacy (SVR12 ≥ 90%) of sofosbuvir plus daclatasvir- and velpatasvir-based regimens in clinical trials supports and reinforces their recommendation by guidelines. Other promising regimens comprise grazoprevir + elbasvir + sofosbuvir, and ombitasvir + paritaprevir/ribavirin + sofosbuvir. Newer regimens incorporating pibrentasvir + glecaprevir or grazoprevir + ruzasvir + MK-3682 (uprifosbuvir), offer all-oral, ribavirin-free SVR12 rates consistently greater than 95%. Observational studies report slightly lower overall SVR rates but reflect corresponding clinical trial data in terms of treatments most likely to achieve good responses.

**Conclusions:**

On the basis of SVR12, we established that for treating GT3 infections (i) regimens incorporating newer DAAs are more effective than those comprising older DAAs, and (ii) ribavirin may be of less benefit in newer DAA regimens than in older DAA regimens. The analysis provides evidence that DAA regimens can replace Peg-IFN-based regimens for GT3 infection.

**Electronic supplementary material:**

The online version of this article (10.1186/s12879-017-2820-z) contains supplementary material, which is available to authorized users.

## Background

Hepatitis C is a persistent viral infection of the liver which, if left untreated, may progress to cirrhosis, decompensated cirrhosis and hepatocellular carcinoma. Six known genotypes of hepatitis C virus (HCV) exist, with genotype 1 (GT1) the most predominant in the USA and Europe. Treatment of GT1 infection results in lower treatment responses with pegylated interferon (Peg-IFN) and ribavirin (RBV) compared to genotype 2 (GT2) and genotype 3 (GT3) infection [1–3]. However, the development of first- and second-generation, direct-acting antiviral (DAA) agents has resulted in almost universal improved sustained virological response (SVR) rates for patients with HCV GT1, 2, 4, 5 and 6. As such, the unmet need has now shifted towards the treatment of GT3 infections.

GT3 is the second most common HCV genotype globally, accounting for 18% of all adult HCV infections [4]. Despite originally being grouped therapeutically, there is increasing evidence to suggest that HCV GT2 and GT3 are clinically distinct. Patients with HCV GT3 infection have a greater risk of developing hepatic steatosis, more rapid progression of hepatic fibrosis and cirrhosis, and hepatocellular carcinoma; they are also less responsive to Peg-IFN-based treatment than are patients with HCV GT2 infection [5, 6].

Patients with chronic HCV GT3 thus have relatively aggressive disease, and subgroups are less responsive to currently licensed DAA treatments; improved strategies to treat and manage HCV GT3 are needed. The challenges associated with identifying the most appropriate regimen for this genotype are complicated by the large body of data. Furthermore, considerable heterogeneity exists between the population composition and outcomes of clinical trials, and findings obtained in clinical practice (i.e. as assessed in observational studies and registries) [7]. The robust systematic literature review reported here aims to summarise all the relevant data available to support optimum treatment decisions for patients with GT3 disease. The focus of the review is to assess the clinical effectiveness of licensed antiviral therapies in the treatment of chronic HCV GT3, as well as new, or newly licensed, treatments, comprising glecaprevir (GLE; formerly ABT-493) plus pibrentasvir (PIB; ABT-530), uprisofbuvir (MK-3682), ruzasvir (RZR; MK-8408), and voxilaprevir (VOX; GS-9857). A careful analysis of the optimal use of RBV was also required, given its additional side effects in combination with DAA regimens, and is included here. The review excludes the outcomes of regimens containing alisporivir, albuferon, consensus interferon, and lambda interferon. Reasons for these exclusions included relatively limited data for each treatment, or experimental, unapproved practices reported in the source material.

The previous standard of care for treating HCV GT3 was pegylated interferon (Peg-IFN) + RBV [1–3], but current guidance, e.g. that of the European Association for the Study of the Liver (EASL), recommends either velpatasvir (VEL) + sofosbuvir (SOF) ± RBV or daclatasvir (DCV) + SOF ± RBV as first-line therapy, with RBV administration dependent upon treatment experience and cirrhotic status [8]. Other relevant guidelines include those of the World Health Organization (WHO) and the American Association for the Study of Liver Diseases (AASLD) [9, 10]. Most DAAs were originally developed using GT1 replicon models; full-length GT1 and GT2 HCV genomes capable of recapitulating the complete virus life cycle in vitro have only recently been developed [11]. Perhaps therefore, relative shortcomings in treatment results with GT3 versus GT1 and GT2 were observed with the first protease and NS5A inhibitors. Indeed, GT3 has been recognised as being a more difficult genotype to treat than GT1 or GT2 [12]. Given that the biology of GT3 is different from GT1, with more rapid progression, steatosis, higher rates of cirrhosis and primary liver cancer [5, 6], optimal treatments are required. Our analysis systematically examines current data with the goal of establishing optimal regimens and the necessity (or otherwise) of including RBV for different categories of patients with HCV GT3 infection.

## Methods

### Study identification

A comprehensive search strategy was designed in accordance with the PRISMA guidelines [13] to identify clinical trials or observational studies providing efficacy data on current antiviral therapies for HCV GT3. Published studies that reported SVR rates at 4 weeks (SVR4), 12 weeks (SVR12) and/or 24 weeks (SVR24) post-treatment in patients with, specifically, chronic HCV GT3 infection were identified and retrieved from indexed databases and grey literature sources. Studies comprising a mixture of genotypes that failed to report GT3-specific data were not included.

A search of the literature published in indexed databases (PubMed, Medline In-Process, and Embase) within the last 5 years (February 2011 to May 2016) was conducted using the search strategy presented in Additional file 1: Table S1 (Search terms in PubMed and Medline in Process) and Additional file 2: Table S2 (Search Terms in Embase). The search of indexed literature was supplemented with a search of five non-indexed (grey) literature sources, comprising annual conferences of the AASLD, Asian Pacific Association for the Study of the Liver (APASL), Conference on Retroviruses and Opportunistic Infections (CROI), EASL, and WHO, restricted to a 1-year period (April 2015 to May 2016). A one-year timeframe was chosen since it was assumed likely that key information presented at congresses would be published in manuscript format within a year and would, thereafter, be ascertained by the literature review.

### Eligibility of studies for inclusion

Bibliographic details and abstracts of all citations retrieved by database and grey literature searches were downloaded into EndNote version X7. Any duplicated citations were excluded before first-pass screening. Citations (titles and abstracts) were screened for eligibility by a single reviewer, based on the inclusion and exclusion criteria provided in Table 1. Studies that did not meet the inclusion criteria were checked by a second reviewer, to reduce the possibility of excluding a relevant report. Subgroups of particular interest comprised HCV GT3 patients with cirrhosis, prior treatment failure, and HIV co-infection, although a greater range of patient types was included in the studies identified and from which data were extracted prior to analysis for the review (Tables 1 and 2).

**Table 1:** Specification of population, intervention, comparison and outcomes (PICO)

	Inclusion criteria	Exclusion criteria
Population	• Adult patients (aged >18 years) • Chronically infected with HCV GT3	• Healthy subjects • Patients without chronic HCV infection • Non-GT3 infection or lack of GT3-specific stratification • Studies in animals • In vitro studies
Intervention	• Any antiviral agent against HCV in at least one arm	• Non-antiviral therapy
Comparison	• Randomised controlled trials • Non-randomised controlled trials • Observational studies (including reports of Registry audits)	• Pharmacokinetics studies • Cost-effectiveness studies • Clinical trial registry entry only • Reviews, editorial, letter or comment • Case control studies • Cohort studies
Outcome	• SVR4 • SVR12 • SVR24 • Unspecified SVR	• If SVR is not reported
Language restrictions	• English language only	• Studies published in language other than English are excluded
Date range	• February 2011 to May 2016	• Studies outside this timeframe are excluded

*GT3* genotype 3, *HCV* hepatitis C virus, *SVR* sustained virological response

### Data extraction from citations

Full texts (including congress abstracts, posters and other congress communications) of citations deemed relevant during title and abstract screening were retrieved for second-pass review. These were assessed for eligibility by a single reviewer, with excluded studies checked by a second reviewer. For all eligible studies, data relevant to study design, trial characteristics, patient characteristics, disease characteristics and treatment outcomes were extracted, as detailed in Table 2.

**Table 2:** Data extraction

Study design	
• Study name, author and year of publication • Study design • Study period • Inclusion/exclusion criteria • Country • Settings • HCV treatment regimen • Definition of cirrhosis detection • HCV RNA quantification limit • Population analysed • Attrition and selection bias	
Trial characteristics	
• Type of publication • Type of study • Treatment regimen • Treatment duration • Ribavirin	
Patient characteristics	
• Proportion of men • Ethnicity (Caucasian/Asian) • Body-mass index • GT3 sample size at baseline • Treatment-naïve patients • CKD stages • Dialysis • Renal transplantation • Concomitant treatment with proton pump inhibitor (PPI)	
Disease characteristics	
• HCV RNA • Baseline resistance-associated variants/substitutions (RAV) • Human immunodeficiency virus (HIV) co-infection • Liver transplantation • Cirrhosis (compensated/decompensated) • MELD score • Child-Pugh score • Liver disease severity (F0/F1/F2/F3/F4)	
Treatment outcomes	
• Rates of SVR (SVR4, SVR12, SVR24 and/or unspecified SVR) • 95% confidence interval (calculated from SVR data, where reported) • Rates of relapse after treatment • Type of relapse • Treatment discontinuation • Serious adverse events (SAEs)	

*CKD* chronic kidney disease, *GT3* genotype 3, *HCV* hepatitis C virus, *MELD* model for end-stage liver disease, *SVR* sustained virological response

Overlapping reports (multiple citations describing a single study) were identified based on matching study names and/or trial numbers, settings, and authors. Sample sizes, years of data collection, and other study characteristics were compared between each citation, to select the most complete and informative data available for extraction. Data were extracted by a single reviewer into Microsoft Excel tables, which were checked by a second reviewer. Reviewers completed data extraction for all fields and noted where data in the relevant field was not reported.

### Analysis of extracted data

For the purposes of this systematic review, analysis of the extracted data focused on a subset of the data containing current treatments tested against HCV GT3 in phase 2 and phase 3 trials and/or real-world studies. These treatments comprised licensed DAAs, including daclatasvir (DCV), elbasvir (EBR), grazoprevir (GZR), pegylated interferon (Peg-IFN), ledipasvir (LDV), ombitasvir (OBV), sofosbuvir (SOF) or SOF-containing, velpatasvir (VEL), as well as new, unlicensed treatments applicable to HCV GT3, including glecaprevir (GLE), pibrentasvir (PIB), MK-3682 (uprofosbuvir), ruzasvir (RZR), and voxilaprevir (VOX). Combinations of these agents with and without RBV, and for all treatment durations except 4 weeks, were also included in the analysis (Table 3). The licensed regimens selected for analysis were those most commonly used in clinical practice. Recently published phase 3 abstract data, beyond the cutoff date (e.g. November 2016, AASLD), could not be included. Although this review focuses on licensed and future treatments, data from regimens used for unlicensed durations (e.g. DCV + SOF for 12 weeks in cirrhotic patients, or SOF + RBV for 12 weeks) were permitted, since such use reflects the reality of clinical practice.

**Table 3:** Treatment regimens analysed among the eligible studies

Treatment regimens	Treatment regimen subgroups
Containing PIB	PIB + GLE
	PIB + GLE + RBV
	PIB + PTV/r + RBV
Containing DCV	DCV + SOF
	DCV + SOF + RBV
Containing GZR	GZR + RZR + MK-3682
	GZR + EBR + MK-3682
	GZR/EBR + SOF
Containing Peg-IFN	Peg-IFN + RBV
	Peg-IFN + SOF + RBV
Containing LDV	LDV + SOF
	LDV + SOF + RBV
Containing OBV	OBV + PTV/r
	OBV + PTV/r + RBV
	OBV + PTV/r + SOF
	OBV + PTV/r + SOF + RBV
Containing SOF	SOF + RBV
Containing VEL	VEL100 + SOF
	VEL100 + SOF + VOX
	VEL100 + SOF + RBV

*DCV* daclatasvir, *EBR* elbasvir, *GLE* glecaprevir, *GZR* grazoprevir, *LDV* ledipasvir, *OBV* ombitasvir, *Peg-IFN* pegylated interferon, *PIB* pibrentasvir, *PTV* paritaprevir, *RBV* ribavirin, *r* ritonavir, *SOF* sofosbuvir, *RZR* ruzasvir, *VEL100* velpatasvir 100 mg, *VOX* voxilaprevir

### Statistical methodology

Where only two of the three SVR parameters (number followed up, number achieving SVR, percentage achieving SVR) were reported, the remaining value was imputed arithmetically prior to any reporting or analysis. Where SVR12 was not available, SVR24 was used; SVR4 was incorporated into the full dataset but not used in the analysis for this review. Treatments were grouped into categories; treatment arms were pooled by category within each study prior to analysis at each level of detail. Pooled estimates and confidence intervals (CIs) were produced using the *metaprop* function within the R meta package (http://meta-analysis-with-r.org//), using the Freeman-Tukey double arcsine transformation [14], which performs well for percentages approaching either 0 or 100 [15]. Heterogeneity across studies was quantified using the I^2^ statistic [16]; a value of 0% indicates no observed heterogeneity, and larger values show increasing heterogeneity [17]. The outputs supporting the forest plots include individual CIs using the Clopper-Pearson method [18], which is the default method in the package used.

## Results

### Studies identified

**Fig. 1:**
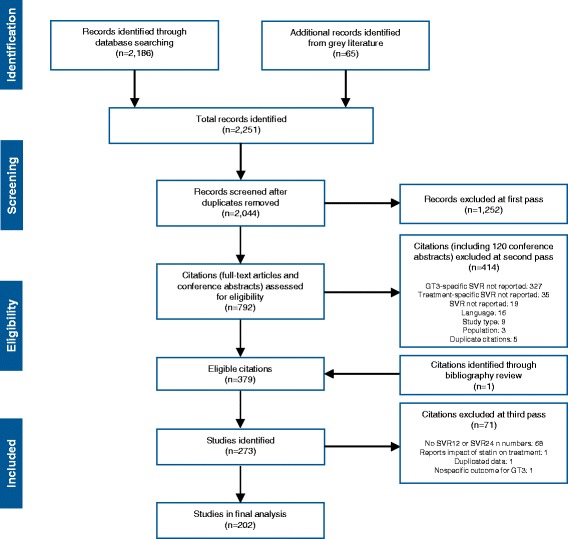
PRISMA flow diagram of study identification. GT3, genotype 3; SLR, systematic literature review; SVR, sustained virological response

Using the search strategies outlined in the Additional file 1: Table S1 and Additional file 2: Table S2, a total of 2186 publications were identified through indexed databases, and an additional 65 citations were retrieved from grey literature sources. Following the removal of duplicate citations and first-pass screening of titles and abstracts, 792 publications were retrieved for full text review. Of these, 413 were excluded during second-pass screening; the primary reason for exclusion was that SVR rates in a HCV GT3-specific population were not reported. Studies which did not report GT3 cohort size were also excluded. One citation was identified from the bibliography of a published systematic review [19, 20] and considered eligible; therefore, a total of 379 publications were included in this study. The number of citations included at each stage of the systematic review is shown in Fig. 1, and a complete list of eligible citations is provided as Additional file 3: List 1. The 379 eligible publications reported SVR data for a wide range of regimens in 273 prospective and retrospective studies, comprising 76 clinical trials and 194 real-world datasets. Where different analyses of the same data were presented in abstracts from different congresses, the most complete dataset was used (this approach led to third-pass exclusion of duplicated data).

The 379 publications reported SVR data for HCV GT3 patients within 721 different study cohorts, treatment arms and/or patient subpopulations. The baseline sample size of HCV GT3 patients reported in these cohorts ranged from 1 to 2106 patients, and baseline patient characteristics were heterogeneous across cohorts. Excluding specific subgroup analyses, baseline characteristics for studies varied substantially for proportions of male (20 to 96%), Caucasian (38 to 98%) and Asian (2 to 58%) patients enrolled. Where reported, body-mass index ranged from 21.3 to 49 kg/m^2^ (mean or median) and HCV RNA viral load ranged between2.03 to 7.5 log_10_ IU/mL (mean or median) across treatment arms, subpopulations and/or whole study across cohorts. In addition to mixed populations, eligible studies also reported SVR data for one or more of the following patient subpopulations: treatment-naïve (272 cohorts); treatment-experienced (121 cohorts); non-cirrhotic (147 cohorts); cirrhotic (127 cohorts); prior liver transplant (27 cohorts); HIV co-infection (49 cohorts). In this analysis, cohorts were considered non-cirrhotic if 0% of patients were cirrhotic. However, it should be noted that cirrhosis was heterogeneously defined across the studies analysed.

### SVR rates by regimen: clinical trial and real-world data

**Fig. 2:**
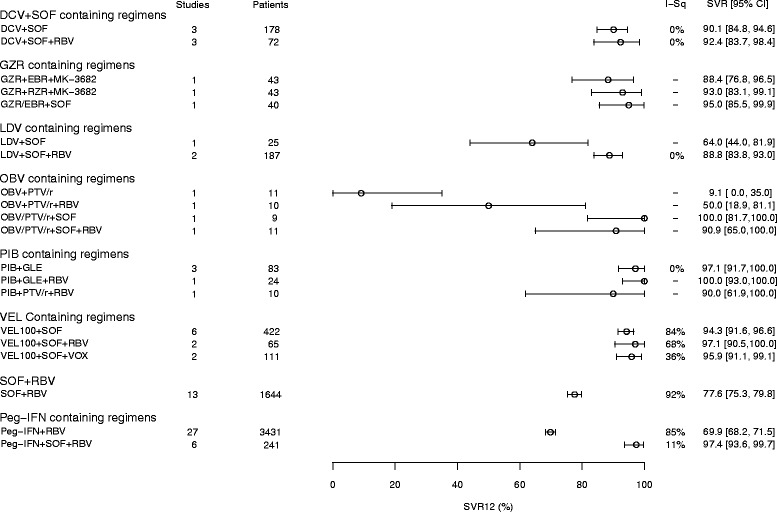
Pooled SVR12 rates for 8 regimens – clinical trials. CI, confidence interval; DCV, daclatasvir; EBR, elbasvir; GLE, glecaprevir; GZR, grazoprevir; I-Sq, I^2^; LDV, ledipasvir; OBV, ombitasvir; Peg-IFN, pegylated interferon; PIB, pibrentasvir; PTV, paritaprevir; RBV, ribavirin; r, ritonavir; SOF, sofosbuvir, RZR, ruzasvir; VEL100, velpatasvir 100 mg; VOX, voxilaprevir. An I^2^ value of 0% indicates no observed heterogeneity; a dash (−) indicates where data are from a single study

**Fig. 3:**
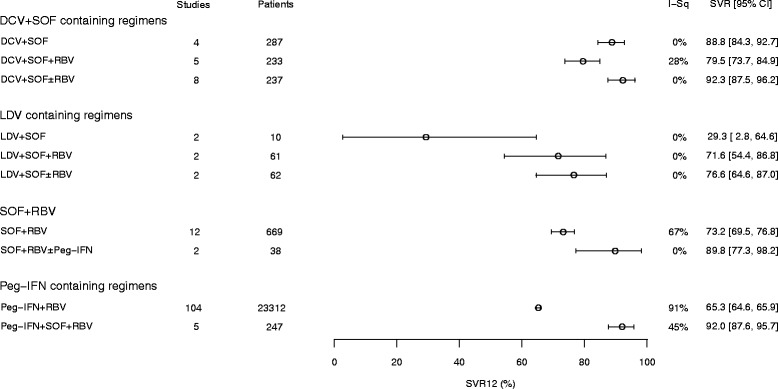
Pooled SVR12 rates for 4 regimens – real-world data. CI, confidence interval; DCV, daclatasvir; I-Sq, I^2^; LDV, ledipasvir; Peg-IFN, pegylated interferon; RBV, ribavirin; SOF, sofosbuvir; SVR12, sustained virological response at 12 weeks. An I^2^ value of 0% indicates no observed heterogeneity

Figures 2 and 3 show forest plots of the pooled SVR rates for the eight clinical trial regimens and the four observational study (real-world) regimens analysed, respectively.

The forest plot of clinical trial data does not include any stratification into patient subgroups (please see Table 4 and Additional file 4: Figure S1, Additional file 5: Figure S2 and Additional file 6 **:** Figure S3 for SVR12 stratification by patient subgroup) but, purely in terms of SVR12 ≥ 90%, of the all-oral regimens, the efficacy of the DCV- and VEL-based regimens supports and reinforces their recommendation by guidelines. Other potentially promising regimens include GZR + EBR + SOF or OBV + PTV/r + SOF. Importantly, PIB + GLE and SOF + VEL + VOX have received marketing authorization and offer SVR12 rates consistently higher than 95%. The GZR + RZR + MK-3682 combination was included as in development at the time of the analysis, although development was halted on 29 September 2017 [21]. The triple-regimen of Peg-IFN + SOF + RBV appears to be a viable interferon-containing option in HCV GT3, but is precluded in increasing numbers of patients. Protease inhibitor-based regimens would also be precluded in patients with decompensated cirrhosis.

Real-world treatment outcomes are reported in significant numbers of patients for the DCV + SOF, Peg-IFN, and SOF + RBV treatments. Although slightly lower overall SVR rates are reported, the results reflect corresponding clinical trial data (Fig. 2) in terms of which treatments are most likely to achieve good responses. In the cases of DCV + SOF ± RBV and Peg-IFN + SOF + RBV, the SVR rates in the real-world studies also seem to approach the rates seen in clinical trials. DCV-containing regimen studies and LDV-containing regimen studies contain many patients from compassionate use/early access programmes, who tended to have more severe liver disease. This could explain some of the differences seen between the SVR rates reported in clinical trial and real-world datasets for the LDV-containing regimen.

### Sub-analyses of clinical trial and observational study regimens

Table 4 and Additional file 4: Figure S1, Additional file 5: Figure S2 and Additional file 6: Figure S3 show patient subgroup stratifications for each of the regimens presented in the clinical-trial forest plot (Fig. 2).

**Table 4:** SVR12 values^a^ for clinical-trial regimens with and without RBV in patients who were (i) cirrhotic or non-cirrhotic^b^, (ii) treatment-experienced or -naïve, or (iii) HIV-positive or -negative

Regimen		SVR12% [95% CI]					
		Cirrhotic	Non-cirrhotic	Treatment-experienced	Treatment-naïve	HIV-positive	HIV-negative
DCV + SOF containing regimens							
DCV + SOF	+RBV	87.3 [74.1, 96.9] *n* = 42	95.5 [76.0, 100.0] *n* = 16	NR	100.0 [68.3, 100.0] *n* = 5	NR	100.0 [68.3, 100.0] *n* = 5
	−RBV	62.5 [44.9, 78.6] *n* = 32	97.4 [93.2, 99.8] *n* = 136	90.8 [79.6, 98.4] *n* = 55	91.7 [87.9, 94.9] *n* = 328	92.3 [69.9, 100.0] n = 13	89.6 [84.1, 94.2] *n* = 165
GZR containing regimens							
GZR + EBR + MK-3682	+RBV	NR	NR	NR	NR	NR	NR
	−RBV	NR	88.4 [76.8, 96.5] *n* = 43	NR	88.4 [76.8, 96.5] *n* = 43	NR	88.4 [76.8, 96.5] *n* = 43
GZR + RZR + MK-3682	+RBV	NR	NR	NR	NR	NR	NR
	−RBV	NR	93.0 [83.1, 99.1] *n* = 43	NR	93.0 [83.1, 99.1] *n* = 43	NR	93.0 [83.1, 99.1] *n* = 43
GZR + EBR + SOF	+RBV	NR	NR	NR	NR	NR	NR
	−RBV	90.9 [65.0, 100.0] *n* = 11	96.6 [85.8, 100.0] *n* = 29	NR	95.0 [85.5, 99.9] *n* = 40	NR	95.0 [85.5, 99.9] *n* = 40
LDV containing regimens							
LDV + SOF	+RBV	79.1 [68.3, 88.3] *n* = 67	93.2 [87.0, 97.7] *n* = 98	82.0 [70.0, 91.6] *n* = 50	91.3 [87.3, 94.7] *n* = 246	NR	88.2 [79.8, 94.6] *n* = 76
	−RBV	NR	NR	NR	64.0 [44.0, 81.9] *n* = 25	NR	64.0 [44.0, 81.9] *n* = 25
OBV containing regimens							
OBV + PTV/r	+RBV	NR	50.0 [18.9, 81.1] *n* = 10	NR	50.0 [18.9, 81.1] *n* = 10	NR	50.0 [18.9, 81.1] *n* = 10
	−RBV	NR	9.1 [0.0, 35.0] *n* = 11	NR	9.1 [0.0, 35.0] *n* = 11	NR	9.1 [0.0, 35.0] *n* = 11
OBV + PTV/r + SOF	+RBV	NR	90.9 [65.0, 100.0] *n* = 11	NR	NR	NR	NR
	−RBV	NR	100.0 [81.7, 100.0] *n* = 9	NR	NR	NR	NR
PIB containing regimens							
PIB + GLE	+RBV	100.0 [93.0, 100.0] *n* = 24	NR	NR	100.0 [93.0, 100.0] *n* = 24	NR	100.0 [93.0, 100.0] *n* = 24
	−RBV	100.0 [93.0, 100.0] *n* = 24	94.6 [88.5, 98.7] *n* = 89	66.7 [5.9, 100.0] *n* = 3	98.0 [92.9, 100.0] *n* = 80	NR	97.1 [91.7, 100.0] *n* = 83
PIB + PTV/r	+RBV	NR	90.0 [61.9, 100.0] *n* = 10	NR	90.0 [61.9, 100.0] *n* = 10	NR	90.0 [61.9, 100.0] *n* = 10
	−RBV	NR	NR	NR	NR	NR	NR
VEL containing regimens							
VEL100 + SOF	+RBV	93.3 [82.2, 99.7] *n* = 39	100.0 [93.5, 100.0] *n* = 26	98.1 [91.9, 100.0] *n* = 52	NR	NR	98.1 [91.9, 100.0] *n* = 52
	−RBV	84.7 [77.8, 90.6] *n* = 132	97.6 [95.1, 99.4] *n* = 278	92.1 [86.4, 96.4] *n* = 124	97.3 [94.6, 99.2] *n* = 260	91.7 [67.6, 100.0] n = 12	93.9 [87.2, 98.5] *n* = 80
VEL100 + SOF + VOX	+RBV	NR	NR	NR	NR	NR	NR
	−RBV	92.9 [83.9, 98.8] *n* = 55	100.0 [92.0, 100.0] *n* = 21	98.7 [92.7, 100.0] *n* = 54	94.4 [86.1, 99.4] *n* = 57	NR	NR
SOF containing regimens							
SOF	+RBV	70.1 [65.9, 74.0] *n* = 540	82.8 [80.4, 85.18] *n* = 1039	77.2 [73.0, 81.2] *n* = 430	81.1 [79.0, 83.1] *n* = 1453	80.4 [75.5, 84.8] *n* = 283	58.5 [53.2, 63.6] *n* = 344
	−RBV	NR	NR	NR	NR	NR	NR
Peg-IFN containing regimens							
Peg-IFN	+RBV	43.6 [35.1, 52.4] *n* = 140	75.4 [69.3, 81.2] *n* = 256	NR	69.1 [67.4, 70.9] *n* = 2928	55.3 [43.0, 67.3] *n* = 69	64.0 [61.6, 66.4] *n* = 1752
	−RBV	NR	NR	NR	NR	NR	NR
Peg-IFN + SOF	+RBV	94.3 [90.0, 97.7] *n* = 201	94.0 [87.4, 98.7] *n* = 119	70.9 [63.7, 77.7] *n* = 170	99.1 [94.8, 100.0] *n* = 133	100.0 [30.3, 100.0] *n* = 2	83.3 [65.4, 96.0] *n* = 24
	−RBV	NR	NR	NR	NR	NR	NR

*CI* confidence interval, *DCV* daclatasvir, *EBR* elbasvir, *GLE* glecaprevir, *GZR* grazoprevir, *I-Sq* I^2^, *LDV*, ledipasvir, *NR* not reported, *OBV* ombitasvir, *Peg-IFN* pegylated interferon, *PIB* pibrentasvir, *PTV* paritaprevir, *RBV* ribavirin, *r* ritonavir, *SOF* sofosbuvir, *RZR* ruzasvir, *VEL100* velpatasvir 100 mg, *VOX* voxilaprevir

^a^Where SVR12 was not available, SVR24 was used

^b^Cohorts were considered non-cirrhotic if 0% of patients were cirrhotic. Where SVR12 values of 100% are presented, this may be due to clinical trials that were exclusively HIV-positive or -negative, exclusively treatment-experienced or -naïve, or exclusively cirrhotic or non-cirrhotic

As expected, the addition of RBV to combinations of SOF and an NS5A inhibitor seems to improve SVR rates in patients on LDV and in cirrhotic patients on VEL + SOF or DCV + SOF. There appears to be a gap in knowledge of outcomes when the new GZR regimens are used with RBV. The discrepancy between the SOF + RBV HIV-positive and -negative patients was probably due to differences in the treatment duration between the two groups; studies which reported SVR rates by HIV status tended to be 12-week treatment duration for HIV-negative patients (e.g. FUSION, POSITRON, FISSION) [22, 23] and 24-week treatment duration for HIV-positive patients (e.g. PHOTON 1, PHOTON 2) [24]. PHOTON 1 had HIV/HCV co-infected patients in 12- and 24-week arms, with the patients in the 24-week arms achieving SVR rates of higher than 83% compared to higher than 63% in the 12-week arm.

Table 5 and Additional file 7: Figure S4, Additional file 8: Figure S5 and Additional file 9: Figure S6 show patient subgroup stratifications for each of the regimens presented in the forest plot of real-world data (Fig. 3).

**Table 5:** SVR12 values for observational study regimens with and without RBV in patients who were (i) cirrhotic or non-cirrhotic ^a^, (ii) treatment-experienced or -naïve, or (iii) HIV-positive or -negative

Regimen		SVR12% [95% CI]					
		Cirrhotic	Non-cirrhotic	Treatment-experienced	Treatment-naïve	HIV-positive	HIV-negative
DCV + SOF containing regimens							
DCV + SOF	+RBV	77.3 [70.9, 83.2] *n* = 186	83.3 [41.4, 100.0] *n* = 6	74.4 [66.7, 81.6] *n* = 139	100.0 [86.1, 100.0] *n* = 12	100.0 [73.9, 100.0] *n* = 6	NR
	−RBV	85.4 [79.7, 90.3] *n* = 215	98.1 [92.2, 100.0] n = 54	81.1 [65.3, 93.5] *n* = 35	90.5 [73.4, 99.8] *n* = 21	97.7 [82.5, 100.0] *n* = 20	NR
	±RBV	84.8 [33.3, 100.0]^b^ *n* = 5	NR	100.0 [73.2, 100.0]^b^ *n* = 6	NR	NR	90.9 [65.0, 100.0]^b^ *n* = 11
LDV containing regimens							
LDV + SOF	+RBV	64.9 [52.0, 76.8] *n* = 57	NR	70.9 [54.0, 86.0] *n* = 63	NR	NR	NR
	−RBV	40.0 [1.8, 86.2] *n* = 5	NR	28.6 [1.0, 68.2] *n* = 7	NR	NR	NR
SOF containing regimens							
SOF	+RBV	49.3 [40.8, 57.7] *n* = 144	81.9 [74.7, 88.2] *n* = 138	60.5 [50.2, 70.4] *n* = 105	74.3 [66.4, 81.5] *n* = 144	100.0 [30.3, 100.0] *n* = 2	NR
	−RBV	NR	NR	NR	NR	NR	NR
SOF ± Peg-IFN	+RBV	90.9 [74.5, 99.8] *n* = 22	NR	91.7 [67.6, 100.0] *n* = 12	NR	NR	NR
	−RBV	NR	NR	NR	NR	NR	NR
Peg-IFN containing regimens							
Peg-IFN	+RBV	63.9 [60.0, 67.7] *n* = 713	77.8 [74.8, 80.7] *n* = 845	49.4 [43.1, 55.7] *n* = 257	63.3 [62.6, 64.1] *n* = 16,031	66.4 [63.1, 69.6] *n* = 874	63.7 [62.8, 64.6] *n* = 11,995
	−RBV	NR	NR	NR	NR	NR	NR
Peg-IFN + SOF	+RBV	100.0 [30.3, 100.0] *n* = 2	NR	NR	NR	NR	NR
	−RBV	NR	NR	NR	NR	NR	NR

*DCV* daclatasvir, *IFN* interferon, *LDV* ledipasvir, *NR* not reported, *RBV* ribavirin, *r* ritonavir, *SOF* sofosbuvir, *SVR12* sustained virological response at 12 weeks

^a^In this analysis, cohorts were considered non-cirrhotic if 0% of patients were cirrhotic

^b^Data from study(s) in which stratification by RBV use was not recorded

The positive effect seen in clinical trials for adding RBV to DCV + SOF in cirrhotic patients is not seen in the real-world studies. This finding probably reflects the fact that the duration of treatment was often 24 weeks in the real-world studies versus 12 weeks in the trials, with the addition of RBV having a larger impact on SVR when the duration of treatment was shorter. The LDV data are from the UK early access programme, which was made up mainly of patients with decompensated cirrhosis.

## Discussion

A recent, large-scale systematic review estimated that, globally, GT1 accounts for 49% of all adult HCV infections, followed by GT3 (18%), GT4 (17%), GT2 (11%), GT5 (2%) and GT6 (1%). However, substantial regional variations in rates occur; GT3 is most prevalent in South Asia (67%) with rates of 54 and 79% seen in India and Pakistan, respectively. GT3 also has high prevalence in Australasia (36%), Tropical Latin America (30%), and Western Europe (29%) [4]. These rates indicate that HCV GT3 infection affects substantial numbers of people and demands attention.

Our detailed analysis supports the current evidence-based guidelines (e.g. EASL [8]) that SOF + DCV, and SOF + VEL, are effective treatments for GT3, having been studied with and without RBV for various durations. However, we found very few reports to compare outcomes after 12 weeks versus 24 weeks (more specifically, 12 weeks with RBV vs. 24 weeks without RBV). Not all the studies were informative: the comparison between 12 and 24 weeks of DCV + SOF was diminished by 12-week + RBV data being derived predominantly from clinical trials, whereas the 24-week data was ascertained predominantly from real-world studies in more advanced, cirrhotic patients. A single 24-week study of SOF + LDV ± RBV did not stratify results by RBV use, and there was only one 24-week SOF + VEL study arm in the review dataset (ASTRAL-4, without RBV) [25]. Since the latter study was done in decompensated cirrhotic patients, a comparatively low SVR12 was observed, and thus comparison with 12-week + RBV data would provide few, if any, meaningful insights. Given the expense of 24-week treatment without RBV in comparison to 12-week treatment without RBV, it is unlikely that gaps in the data will be addressed as newer treatments come along that might be successful without RBV.

In our analysis, cirrhosis and decompensated cirrhosis were considered as a unit (whereas cirrhosis encompasses a heterogenous spectrum of stages once portal hypertension and oesophageal varices become evident; relatively few data were available for decompensated cirrhosis). Hence, optimal regimens for patients with less advanced cirrhosis, cirrhosis with evident portal hypertension, and cirrhosis with decompensated liver disease could not be determined with any certainty. However, decompensated patients generally do not fare as well as patients with Child-Pugh A cirrhosis. In 10 eligible studies, SVRs in decompensated cirrhosis ranged from 40 to 88%, irrespective of regimen/duration (data not shown). Nonetheless, it remains important to treat a large residual cohort of decompensated cirrhotic patients worldwide because of (i) the possibility of amelioration of the liver disease after a cure, and (ii) the absence of liver transplantation as an option for most patients, before elimination of infection and disease can be envisaged (given the cost of drugs and potential scale of undiagnosed patient populations).

Within the time-frame constraints of this systematic review, we were unable to compare recently abstracted but now licensed regimens for GT3, including SOF + VEL + VOX (POLARIS-3) [26], PIB + GLE for 12 or 16 weeks in HCV GT3 and cirrhosis (SURVEYOR II) [27], or SOF + GZR + EBR (C-ISLE) [28]; however, response rates were favourable, ranging from 91 to 100%, suggesting that new, second wave, or double or triple regimens will bring treatment of GT3 to parity with outcomes for other genotypes.

RBV seems to have less of an impact in improving SVR12 rates when incorporated into the newest regimens (comprising PIB + GLE, VEL + SOF, and DCV + SOF) than as a component of older regimens. RBV had a particularly large effect on improving SVR12 rate when added to OBV + PTV/r + SOF, although overall effectiveness remained poor (SVR12 of 50%). SVR12 rates for SOF + RBV seem similar to those for Peg-IFN + RBV, but SVR12 for IFN + SOF + RBV was 17% lower in HIV-negative (83%) than in HIV-positive patients (100%), and substantially lower for SOF + RBV in HIV-negative versus HIV-positive patients (58.5% vs. 80.4%). However, these RBV-related, HIV status-stratified differences may be due to variation in study design and/or statistical fluke, and no hard conclusions can be drawn from the results. In one small study, RBV addition was also detrimental to SVR12 when added to OBV + PTV/r + SOF, causing a 10% reduction in the rate, suggesting that further studies might be needed in some of the newer regimens still under investigation.

Slightly lower SVR12 rates observed in the real-world data are likely to reflect factors such as greater patient heterogeneity and poorer treatment adherence in the real-world versus clinical trials, exclusion of high-risk groups, and overall better level of care in clinical trials as compared with that in actual clinical practice. It should also be noted that, during the period of data identification/extraction, physicians tended to have prioritised treatment of patients with more advanced liver disease, who are known to be less likely to achieve SVR. Therefore, observational studies in this period would have been weighted to include patients who were more difficult to treat. Some real-world data were obtained from several compassionate use programmes, which focused on patients with advanced disease, including those with decompensated cirrhosis.

The strengths of this review include a rigorous implementation of the PRISMA approach, the large number of citations identified/analysed, systematic and rational reasons for exclusion of citations, breakdown of critical factors (e.g. RBV use, inclusion of subgroups – HIV co-infection, cirrhosis, treatment experience), and comprehensive, robust statistical analysis of the extracted data. The inclusion of both real-world data and clinical trial data allows demonstration that real-world data support the findings of clinical trials, confirming the trend towards higher SVR12 rates with newer DAAs, and the role of RBV in all the regimens analysed.

This review is limited in being tightly focused on effectiveness; as such, it does not assess safety, the impact of baseline resistance, quality of life, or any other long-term factors. We were unable to compare efficacy against GT3 subtypes or GT3 emanating from the Asian sub-continent or elsewhere; genotypic, phenotypic and host responses may differ. The number of studies eligible for analysis was also small (1–2 studies) in some instances, e.g. for OBV-, LDV-, and GZR-containing regimens. However, the field of HCV treatment is a dynamic and constantly changing landscape: a number of agents that were unlicensed at the time of this analysis are now available, and for others development has ceased [21, 29].

Owing to necessary grouping of subgroups of interest (HIV co-infection, cirrhosis, treatment experience), difficulties emerged upon analysis; for example, out of necessity, we had to extract data from clinical trials or real-world studies that grouped patients with GT3 together with patients infected with other HCV genotypes. More recently, clinical trials have restricted inclusion to patients with only GT3 -- for example, the POLARIS 3, ENDURANCE 3, SURVEYOR 2, ASTRAL-2, and ASTRAL-3 studies. However, the present analysis nevertheless provides a comprehensive and careful analysis of the efficacy of current DAA regimens for GT3, and draws attention to the availability of interferon-free regimens for this group of patients. Another challenge was the definition of cirrhosis, which was heterogeneous in the studies included, with both invasive and non-invasive methodologies used. This resulted in a wide spectrum of cirrhotic states/severity in our dataset, which is likely to have impacted upon SVR12 rates. Another population largely absent from our dataset was GT3 patients with renal disease and/or renal failure represent a population that the review dataset lacks; more recent studies have reported on this population [30–38],

From a statistical perspective, the outputs supporting the forest plots include individual CIs determined using the Clopper-Pearson method [18], which is the default method in the *metaprop* package used. However, because individual CIs were not a component of the deliverables of the pooled analysis, it was not investigated whether this constituted a good choice for percentages approaching 100, which were observed for many SVR12 rates, particularly among the newer regimens.

Guidelines for HCV management reflect a consensus of expert opinion informed by evidence and data, but they are not necessarily developed based on rigorous, systematic review, hence this attempt to bridge the gap. It will be the place of national and international scientific societies to provide updated guidelines. However, in our analysis, encouraging efficacy results for GT3 infection were observed for several new DAA regimens, including SOF + DCV, SOF + VEL, SOF + VEL + VOX, PIB + GLE, and GZR + RZR + MK-3682 (although the latter has now been withdrawn [21]). These data provide conclusive evidence to guide recommendations and sugges that there is no longer a need for interferon-based regimens for GT3. We anticipate that our results will help national and international scientific societies to continue to make dynamic and firm recommendations for the optimum use of DAA regimens, with and without ribavirin, in both treatment naïve and treatment-experienced GT3 patients with and without cirrhosis [39].

## Conclusions

This systematic analysis provides a detailed comparison of DAA regimens with improved potency against GT3 hepatitis C, including those incorporating guideline-recommended and as yet unlicensed DAAs. These regimens improve SVR rates overall for this genotype and deliver an evolution of treatment for patients with HCV GT3. Notwithstanding the efficacy of SOF + Peg-IFN + RBV, our review of the data provides evidence that this Peg-IFN-based regimen can be effectively superseded by DAA regimens for GT3 treatment.

Whether RBV should be added to regimens containing PIB + GLE or SOF + VEL (and the optimal duration) for treatment-experienced cirrhotic patients, or to salvage regimens for retreatment of patients with NS5A resistance-associated substitutions remains an open question. We did not review efficacy for retreatment in this review because such analysis would require a separate systematic review. Most of the DAA regimens studied in the period covered by the present review were used for patients who had failed pegylated interferon and ribavirin, sofosbuvir, or sofosbuvir and ribavirin; the data for retreatment of GT-3 NS5A, protease or NS5B inhibitors are only now maturing. Several trials were published after the cut-off date for this analysis, but as late as May 2017, there were no approved rescue regimens for such patients. Trials of newer combinations such as SOF + VEL, SOF + VEL + VOX, GLE + PIB, GLE + PIB + SOF are in progress, but development of GZR + RZR + MK-3682, GZR + RZR + SOF, and AL-335 + odalasvir + SIM has been halted [1, 21, 29, 40–50].

## Additional files

Additional file 1: Table S1. Search terms in PubMed (Medline in Process). Table summarising search terms used in PubMed and Medline in Process. (DOCX 13 kb)

Additional file 2: Table S2. Search terms in Embase. Table summarising search terms used in Embase. (DOCX 13 kb)

Additional file 3: **List 1.** Eligible citations from systematic literature review. List of the 379 publications identified in systematic literature review that reported SVR data and were eligible for inclusion in this analysis. (DOCX 59 kb)

Additional file 4: Figure S1. Clinical trial pooled SVR12 rates for (a) patients with cirrhosis and (b) patients without cirrhosis. Forest plot showing SVR12 rates from clinical trials stratified by the presence or absence of cirrhosis. (PDF 11 kb)

Additional file 5: Figure S2. Clinical trial pooled SVR12 rates for (a) treatment-experienced patients and (b) treatment-naïve patients. Forest plot showing SVR12 rates from clinical trials stratified by previous treatment history. (PDF 10 kb)

Additional file 6: Figure S3. Clinical trial pooled SVR12 rates for (a) patients without HIV and (b) patients with HIV. Forest plot showing SVR12 rates from clinical trials stratified by the presence or absence of HIV. (PDF 10 kb)

Additional file 7: Figure S4. Real-world pooled SVR12 rates for (a) patients with cirrhosis and (b) patients without cirrhosis. Forest plot showing SVR12 rates from real-world datasets stratified by the presence or absence of cirrhosis. (PDF 22 kb)

Additional file 8: Figure S5. Real world pooled SVR12 rates for (a) treatment-experienced patients and (b) treatment-naïve patients. Forest plot showing SVR12 rates from real-world datasets stratified by previous treatment history. (PDF 31 kb)

Additional file 9: Figure S6. Real world pooled SVR12 rates for (a) patients without HIV and (b) patients with HIV. Forest plot showing SVR12 rates from real-world datasets stratified by the presence or absence of HIV. (PDF 9 kb)

## Abbreviations

AASLD: American Association for the Study of Liver Diseases
APASL: Asian Pacific Association for the Study of the Liver
CI: Confidence interval
CKD: Chronic kidney disease
CROI: Conference on Retroviruses and Opportunistic Infections
DAA: Direct-acting antiviral
DCV: Dacalatasvir
EASL: European Association for the Study of the Liver
EBR: Elbasvir
GLE: Glecaprevir
GT1: Genotype 1
GT2: Genotype 2
GT3: Genotype 3
GT4: Genotype 4
GT5: genotype 5
GT6: Genotype 6
GZR: Grazoprevir
HCV: Hepatitis C virus
HIV: Human immunodeficiency virus
I-Sq: I^2^ – percentage of variation across studies that is due to heterogeneity rather than chance
LDV: Ledipasvir
MELD: Model for end-stage liver disease
NS5A: Nonstructural protein 5A
OBV: Ombitasvir
Peg-IFN: Pegylated interferon
PIB: Pibrentasvir
PPI: Proton pump inhibitor
PRISMA: Preferred reporting items for systematic reviews and meta-analyses
PTV: Paritaprevir
r: Ritonavir
RAV: Resistance-associated variants/substitutions
RBV: Ribavirin
RZR: Ruzasvir
SAE: Serious adverse event
SOF: Sofosbuvir
SVR: Sustained virological response
SVR12: SVR at 12 weeks
SVR24: SVR at 24 weeks
SVR4: SVR at 4 weeks
VEL100: Velpatasvir 100 mg
VOX: Voxilaprevir
WHO: World Health Organization

## References

[1] European Association for the Study of the Liver. EASL recommendations on treatment of hepatitis C 2014. Available from: http://www.easl.eu/medias/EASLimg/News/easl_recommendations_hcv_2014_full.pdf. Accessed Jan 2017. 10.1016/j.jhep.2022.10.006 36464532

[2] National Institute for Health and Care Excellence. Interferon alfa (pegylated and non-pegylated) and ribavirin for the treatment of chronic hepatitis C. NICE technology appraisal guidance [TA75]. 2004. 22 August 2016. Available from: https://www.nice.org.uk/guidance/ta75. Accessed Jan 2017.

[3] National Institute for Health and Care Excellence. Peginterferon alfa and ribavirin for the treatment of chronic hepatitis C. NICE technology appraisal guidance [TA200]. 2010. 15 August 2014. Available from: https://www.nice.org.uk/guidance/TA200. Accessed Jan 2017.

[4] Petruzziello A, Marigliano S, Loquercio G, Cozzolino A, Cacciapuoti A (2016). Global epidemiology of hepatitis C virus infection: an up-date of the distribution and circulation of hepatitis C virus genotypes. World J Gastroenterol.

[5] Andriulli A, Mangia A, Iacobellis A, Ippolito A, Leandro G, Zeuzem S (2008). Meta-analysis: the outcome of anti-viral therapy in HCV genotype 2 and genotype 3 infected patients with chronic hepatitis. Aliment Pharmacol Ther.

[6] Kanwal F, Kramer JR, Ilyas J, Duan Z, El-Serag HB (2014). HCV genotype 3 is associated with an increased risk of cirrhosis and hepatocellular cancer in a national sample of U.S. veterans with HCV. Hepatology.

[7] Wu CJ, Roytman MM, Hong LK, Huddleston J, Trujillo R, Cheung A (2015). Real-world experience with sofosbuvir-based regimens for chronic hepatitis C, including patients with factors previously associated with inferior treatment response. Hawaii J Med Public Health.

[8] European Association for the Study of the Liver. EASL recommendations on treatment of hepatitis C 2016. J Hepatol. 2017;66(1):153–94. 10.1016/j.jhep.2016.09.001. 10.1016/j.jhep.2016.09.001 27667367

[9] World Health Organization. Guidelines for the screening, care and treatment of persons with chronic hepatitis C infection. Updated version April 2016. Available from: http://apps.who.int/iris/bitstream/10665/205035/1/9789241549615_eng.pdf?ua=1. Accessed Jan 2017. 27227200

[10] American Association for the Study of Liver Diseases. Recommendations for testing, managing, and treating hepatitis C. Available from: http://www.hcvguidelines.org/. Accessed Jan 2017.

[11] Catanese MT, Dorner M (2015). Advances in experimental systems to study hepatitis C virus in vitro and in vivo. Virology.

[12] Ampuero J, Romero-Gómez M, Reddy KR (2014). HCV genotype 3 – the new treatment challenge. Aliment Pharmacol Ther.

[13] Moher D, Liberati A, Tetzlaff J, Altmann G (2009). The PRISMA group. Preferred reporting items for systematic reviews and meta-analyses: the PRISMA statement. PLoS Med.

[14] Freeman MF, Tukey JW (1950). Transformations related to the angular and the square root. Ann Math Statist.

[15] Barendregt JJ, Doi SA, Lee YY, Rosana E, Norman RE, Vos T (2013). Meta-analysis of prevalence. J Epidemiol Community Health.

[16] Higgins JPT, Thompson SG (2002). Quantifying heterogeneity in a meta-analysis. Stat Med.

[17] Higgins JPT, Thompson SG, Deeks JJ, Altman DG (2003). Measuring inconsistency in meta-analyses. BMJ.

[18] Clopper CJ, Pearson ES (1934). The use of confidence or fiducial limits illustrated in the case of the ‘binomial. Biometrika.

[19] Swallow E, Song J, Yuan Y, Kalsekar A, Kelley C, Peeples M (2016). Daclatasvir and sofosbuvir versus sofosbuvir and ribavirin in patients with chronic hepatitis C coinfected with HIV: a matching-adjusted indirect comparison. Clin Ther.

[20] Kohli A, Shaffer A, Sherman A, Kottilil S (2014). Treatment of hepatitis C: a systematic review. JAMA.

[21] Merck, 2017. Merck Discontinues MK-3682B and MK-3682C Development Programs. Available from: http://www.mrknewsroom.com/news-release/corporate-news/merck-discontinues-mk-3682b-and-mk-3682c-development-programs. Accessed Oct 2017.

[22] Jacobson IM, Gordon SC, Kowdley KV, Yoshida EM, Rodriguez-Torres M, Sulkowski M (2013). Sofosbuvir for hepatitis C genotype 2 or 3 in patients without treatment options. N Engl J Med.

[23] Lawitz EL, Mangia A, Wyles D, Rodriguez-Torres M, Hassanein T, Gordon SC (2013). Sofosbuvir for previously untreated chonic hepatitis C infection. N Engl J Med.

[24] Younossi ZM, Stepanova M, Sulkowski M, Naggie S, Puoti M, Orkin C (2015). Sofosbuvir and ribavirin for treatment of chronic hepatitis C in patients coinfected with hepatitis C virus and HIV: the impact on patient-reported outcomes. J Infect Dis.

[25] Curry MP, O'Leary JG, Bzowej N, Muir AJ, Korenblat KM, Fenkel JM (2015). Sofosbuvir and velpatasvir for HCV in patients with decompensated cirrhosis. N Engl J Med.

[26] Foster GR, Thompson AJ, Ruane PJ, Borgia S, Dore G, Workowski K, et al. A randomized phase 3 trial of sofosbuvir/velpatasvir/voxilaprevir for 8 weeks and sofosbuvir/velpatasvir for 12 weeks for patients with genotype 3 HCV infection and cirrhosis: The POLARIS-3 Study. 67th Meeting of the American Association for the Study of Liver Diseases: Liver Meeting 2016. Boston, November 11–15, 2016. Abstract 258.

[27] Kwo PY, Wyles DL, Wang S, Poordad F, Gane E, Maliakkal B, et al. 100% SVR12 with ABT-493 and ABT-530 with or without ribavirin in treatment-naïve HCV genotype 3-infected patients with cirrhosis. 51st Annual Meeting of the European Association for the Study of the Liver: Barcelona April 13–17, 2016. Abstract LB01.

[28] Foster GR, Cooper C, Molina J-M, Naggie S, Huang KC, Osinusi A, et al. C-ISLE: Grazoprevir/elbasvir plus sofosbuvir in treatment-naïve and treatment-experienced HCV GT3 cirrhotic patients treated for 8, 12 or 16 weeks. 67th Meeting of the American Association for the Study of Liver Diseases: Liver Meeting 2016. Boston, November 11-15, 2016. Abstract 74.

[29] Janssen, 2017. Janssen to Discontinue Hepatitis C Development Program. Available from: http://www.janssen.com/janssen-discontinue-hepatitis-c-development-program. Accessed Oct 2017.

[30] Bruchfeld A, Roth D, Martin P, Nelson DR, Pol S, Londoño MC (2017). Elbasvir plus grazoprevir in patients with hepatitis C virus infection and stage 4-5 chronic kidney disease: clinical, virological, and health-related quality-of-life outcomes from a phase 3, multicentre, randomised, double-blind, placebo-controlled trial. Lancet. Gastroenterol Hepatol.

[31] Cholongitas E, Pipili C, Papatheodoridis GV (2017). Interferon-free regimens in patients with hepatitis C infection and renal dysfunction or kidney transplantation. World J Hepatol.

[32] Colombo M, Aghemo A, Liu H, Zhang J, Dvory-Sobol H, Hyland R (2017). Treatment with ledipasvir-sofosbuvir for 12 or 24 weeks in kidney transplant recipients with chronic hepatitis C virus genotype 1 or 4 infection: a randomized trial. Ann Intern Med.

[33] Desnoyer A, Pospai D, Lê MP, Gervais A, Heurgué-Berlot A, Laradi A (2016). Pharmacokinetics, safety and efficacy of a full dose sofosbuvir-based regimen given daily in hemodialysis patients with chronic hepatitis C. J Hepatol.

[34] Fabrizi F, Donato FM, and Messa P. Direct-acting antivirals for hepatitis C virus in patients on maintenance dialysis. Int J Artif Organs. 8 July 2017 [epub ahead of print]. doi:10.5301/ijao.5000613 10.5301/ijao.5000613 28708211

[35] Pockros PJ, Reddy KR, Mantry PS, Cohen E, Bennett M, Sulkowski MS (2016). Efficacy of direct-acting antiviral combination for patients with hepatitis C virus genotype 1 infection and severe renal impairment or end-stage renal disease. Gastroenterology.

[36] Reddy KR, Roth D, Bruchfeld A, Hwang P, Haber B, Robertson MN, et al. Elbasvir/grazoprevir does not worsen renal function in patients with hepatitis C virus infection and pre-existing renal disease. Hepatol Res. 23 March 2017 [epub ahead of print]. doi:10.1111/hepr.12899. 10.1111/hepr.12899 28334495

[37] Roth D, Nelson DR, Bruchfeld A, Liapakis A, Silva M, Monsour H (2015). Grazoprevir plus elbasvir in treatment-naive and treatment-experienced patients with hepatitis C virus genotype 1 infection and stage 4-5 chronic kidney disease (the C-SURFER study): a combination phase 3 study. Lancet.

[38] Saxena V, Khungar V, Verna EC, Levitsky J, Brown RS, Hassan MA (2017). Safety and efficacy of current direct-acting antiviral regimens in kidney and liver transplant recipients with hepatitis C: results from the HCV-TARGET study. Hepatology.

[39] AASLD and IDSA. HCV guidance: recommendations for testing, managing, and treating hepatitis C. 2017. Available at: http://hcvguidelines.org/. Accessed Oct 2017.

[40] Wyles D, Wedemeyer H, Ben-Ari Z, Gane EJ, Hansen JB, Jacobson IM, et al. Grazoprevir, ruzasvir, and uprifosbuvir for HCV after NS5A treatment failure. Hepatology. 7 July 2017 [epub ahead of print]. doi:10.1002/hep.29358. 10.1002/hep.29358 28688129

[41] Wyles D, Poordad F, Wang S, Alric L, Felizarta F, Kwo PY, et al. Glecaprevir/pibrentasvir for HCV genotype 3 patients with cirrhosis and/or prior treatment experience: a partially randomized phase III clinical trial. Hepatology. 19 September 2017 [epub ahead of print]. doi:10.1002/hep.29541. 10.1002/hep.29541 PMC5817409 28926120

[42] Voaklander R, Jacobson IM (2017). Sofosbuvir, velpatasvir and voxilaprevir combination for the treatment of hepatitis C. Expert Rev Gastroenterol Hepatol.

[43] Tong L, Yu W, Chen L, Selyutin O, Dwyer MP, Nair AG (2017). Discovery of ruzasvir (mk-8408): a potent, pan-genotype HCV NS5A inhibitor with optimized activity against common resistance-associated polymorphisms. J Med Chem.

[44] Poordad F, Felizarta F, Asatryan A, Sulkowski MS, Reindollar RW, Landis CS (2017). Glecaprevir and pibrentasvir for 12 weeks for hepatitis C virus genotype 1 infection and prior direct-acting antiviral treatment. Hepatology.

[45] Molino S, Martin MT (2017). Hepatitis c virus resistance testing in genotype 1: the changing role in clinical utility. Ann Pharmacother.

[46] Lawitz E, Poordad F, Wells J, Hyland RH, Yang Y, Dvory-Sobol H (2017). Sofosbuvir-velpatasvir-voxilaprevir with or without ribavirin in direct-acting antiviral-experienced patients with genotype 1 hepatitis C virus. Hepatology.

[47] Lawitz E, Buti M, Vierling JM, Almasio PL, Bruno S, Ruane PJ, et al. Safety and efficacy of a fixed-dose combination regimen of grazoprevir, ruzasvir, and uprifosbuvir with or without ribavirin in participants with and without cirrhosis with chronic hepatitis C virus genotype 1, 2, or 3 infection (C-CREST-1 and C-CREST-2, part B): two randomised, phase 2, open-label trials. Lancet Gastroenterol Hepatol. 9 August 2017 [epub ahead of print]. doi:10.1016/S2468-1253(17)30163-2. 10.1016/S2468-1253(17)30163-2 28802814

[48] Gane EJ, Shiffman ML, Etzkorn K, Morelli G, Stedman CAM, Davis MN (2017). Sofosbuvir-velpatasvir with ribavirin for 24 weeks in hepatitis C virus patients previously treated with a direct-acting antiviral regimen. Hepatology.

[49] Bourlière M, Gordon SC, Flamm SL, Cooper CL, Ramji A, Tong M (2017). Sofosbuvir, Velpatasvir, and Voxilaprevir for previously treated HCV infection. N Engl J Med.

[50] Gane EJ, Schwabe C, Hyland RH, Yang Y, Svarovskaia E, Stamm LM (2016). Efficacy of the combination of sofosbuvir, velpatasvir, and the NS3/4A protease inhibitor GS-9857 in treatment-naive or previously treated patients with hepatitis C virus genotype 1 or 3 infections. Gastroenterology.

