# Supplementary material for: Effectiveness of current and future regimens for treating genotype 3 hepatitis C virus infection: a large-scale systematic review
Source: BMC Infect Dis. 2017 Nov 16;17:722. doi: 10.1186/s12879-017-2820-z (PMC5691805; doi:10.1186/s12879-017-2820-z)
Supplement: Supplementary file 1 — Search terms in PubMed (Medline in Process). Table summarising search terms used in PubMed and Medline in Process. (DOCX 13 kb) [file 12879_2017_2820_MOESM1_ESM.docx]

**Additional file 1: Table S1 Search terms in PubMed (Medline in Process)**

| **Line number** | **Search term** | **Hits** |
| --- | --- | --- |
| 1 | ("Hepatitis C"[Mesh] OR "hepatitis C"[TIAB]) | 73,625 |
| 2 | ((((("sustained virologic response") OR "sustained virological response") OR SVR) OR svr4) OR svr12 OR SVR24) | 7,951 |
| 3 | ((genotype AND 3) OR "GT 3" OR gt3 OR g3) | 148,218 |
| 4 | #1 AND #2 AND #3 | 2,366 |
| 5 | ((((((("Case Reports"[Publication Type]) OR "letter"[Publication Type]) OR "editorial"[Publication Type]) AND review[Publication Type]) NOT systematic) OR (meta AND analy*)) OR indirect) OR (mixed AND "treatment comparison") | 397,316 |
| 6 | #4 NOT #5 | 2,243 |
| 7 | #6 Filters: Publication date from 2011/02/01 to 2016/05/18 | 1,370 |
