# Supplementary material for: Effectiveness of current and future regimens for treating genotype 3 hepatitis C virus infection: a large-scale systematic review
Source: BMC Infect Dis. 2017 Nov 16;17:722. doi: 10.1186/s12879-017-2820-z (PMC5691805; doi:10.1186/s12879-017-2820-z)
Supplement: Supplementary file 2 — Search terms in Embase. Table summarising search terms used in Embase. (DOCX 13 kb) [file 12879_2017_2820_MOESM2_ESM.docx]

**Additional file 2: Table S2. Search terms in Embase**

| **Line number** | **Search term** | **Hits** |
| --- | --- | --- |
| 1 | 'hepatitis c'/exp | 86,037 |
| 2 | 'hepatitis c':ab OR 'hcv':ab | 91,081 |
| 3 | 'genotype 3' OR 'gt 3' OR 'gt3' OR g3 | 24,655 |
| 4 | 'sustained virologic response' OR 'sustained virological response' OR svr OR svr12 OR svr4 OR svr24 | 15,144 |
| 5 | #1 OR #2 | 119,761 |
| 6 | #5 AND #3 AND #4 | 1,115 |
| 7 | #5 AND #3 AND #4 ([article]/lim OR [article in press]/lim OR [conference abstract]/lim OR [conference paper]/lim) AND [humans]/lim AND [english]/lim AND [2011-2016]/py | 816 |
