# Supplementary material for: Effectiveness of current and future regimens for treating genotype 3 hepatitis C virus infection: a large-scale systematic review
Source: BMC Infect Dis. 2017 Nov 16;17:722. doi: 10.1186/s12879-017-2820-z (PMC5691805; doi:10.1186/s12879-017-2820-z)
Supplement: Supplementary file 3 — List 1. Eligible citations from systematic literature review. List of the 379 publications identified in systematic literature review that reported SVR data and were eligible for inclusion in this analysis. (DOCX 59 kb) [file 12879_2017_2820_MOESM3_ESM.docx]

**Additional file 3 List 1 Eligible citations from systematic literature review**

1. Abbas Z, Raza S, Hamid S, et al. Randomized controlled trial of interferon gamma versus amantadine in combination with interferon alpha and ribavirin for hepatitis C genotype 3 non-responders and relapsers. J Pak Med Assoc. 2012;62(4):338–43.

2. Abbas Z, Tayyab GN, Qureshi M, et al. Consensus interferon plus ribavirin for hepatitis C genotype 3 patients previously treated with pegylated interferon plus ribavirin. Hepat Mon. 2013;13(12):e14146.

3. Abbas Z, Umer MA, Yakoob J, et al. IL28B, rs12979860 variants do not predict virological response in chronic hepatitis C genotype 3. Hepatol Int. 2014;8(1):S190.

4. Acharya SK, Sreenivas V, Gupta SD, et al. Treatment of chronic hepatitis due to hepatitis C virus (Ch-C) in india: a randomized controlled trial comparing daily interferon-alfa-2b and ribavirin with daily interferon-alfa-2b and glycyrrhizin-a multicenter study. J Clin Exp Hepatol. 2012;2(1):10–8.

5. Afshar P, Nyberg AH, Burchette RJ, et al. Association of low density lipoprotein and sustained virologic response in genotypes 2 and 3 chronic hepatitis c patients treated with peginterferon and ribavirin, a single center study. Gastroenterology. 2012;142(5):S964–5.

6. Afzal-ul-haq Haqqi S, Siddiqui AR, Taj MA, et al. Pegylated interferon in HCV genotype 3 relapsers to conventional interferon in Pakistani population. Hepatol Int. 2016;10(1):S130.

7. Agarwal K, Patel A, Samuel D, et al. SOF/VEL for 12 weeks results in high SVR12 rates in subjects with negative predictors of response to treatment: an integrated analysis of efficacy from the ASTRAL-1, ASTRAL-2 and ASTRAL-3 studies. J Hepatol. 2016;64:S787–8.

8. Agarwala T, Ray G. Factors influencing response of chronic hepatitis C to peginterferon and ribavirin therapy. Indian J Gastroenterol. 2014;33(1):A57.

9. Aguilera V, Rubin A, Prieto M, et al. Baseline pegylated interferon (PIFN) and calcineurin inhibitor (CNI): No impact on sustained viral response (SVR) post-liver transplantation. Liver Transplantation. 2011;17:S97.

10. Ahmed OF, Marra F, Fox R, et al. Sofosbuvir/daclatsavir/ribavirin for 12 weeks in patients with genotype 3 hepatitis C and advanced fibrosis/ cirrhosis: results from a real world cohort. J Hepatol. 2016;64:S804–5.

11. Akkarathamrongsin S, Payungporn S, Thong VD, et al. Early viral kinetics during hepatitis C virus genotype 6 treatment according to IL28B polymorphisms. World J Gastroenterol. 2014;20(30):10599–605.

12. Akkarathamrongsin S, Thong V, Poovorawan Y, et al. Role of interferon lambda-4 and inosine triphosphatase polymorphisms in the treatment of Thai patients with chronic hepatitis C virus genotype 3. J Viral Hepat. 2014;21:30–1.

13. Akkarathamrongsin S, Thong VD, Payungporn S, et al. IFNL3 (IL28B) and IFNL4 polymorphisms are associated with treatment response in Thai patients infected with HCV genotype 1, but not with genotypes 3 and 6. J Med Virol. 2014;86(9):1482–90.

14. Al-Enzi SA, Ismail WA, Alsurayei SA, et al. Peginterferon alfa-2b and ribavirin therapy in Kuwaiti patients with chronic hepatitis C virus infection. East Mediterr Health J. 2011;17(8):669–78.

15. Ali I, Khan S, Attaullah S, et al. Response to combination therapy of HCV 3a infected Pakistani patients and the role of NS5A protein. Virol J. 2011;8:258.

16. Alqahtani S, Zeuzem S, Manns M, et al. Safety and effectiveness of sofosbuvir-based regimens for the treatment of hepatitis C genotype 3 and 4 infections: Interim analysis of a prospective, observational study. J Hepatol. 2015;62:S652–S3.

17. Alqatani S, Zeuzem S, Bourgeois S, et al. On treatment HCV RNA as a predictor of SVR12 in patients with genotype 1–6 HCV infection treated with sofosbuvir/velpatasvir fixed dose combination for 12 weeks: an analysis of the ASTRAL-1, ASTRAL-2, and ASTRAL-3 studies. J Hepatol. 2016;64:S817.

18. Amanzada A, Goralczyk A, Moriconi F, et al. Ultra-rapid virological response, young age, low γ-GT/ALT-ratio, and absence of steatosis identify a subgroup of HCV genotype 3 patients who achieve SVR with IFN-α2a monotherapy. Dig Dis Sci. 2011;56(11):3296–304.

19. Amanzada A, Moriconi F, Lindhorst A, et al. IL28B polymorphism and γ-GT/ALT-ratio as predictive factors for interferon-α2A based treatment response in patients with hepatitis C virus genotype 3 infection. Hepatology. 2011;54:869A.

20. Amir M, Rahman AS, Jamal Q, et al. End treatment response and sustained viral response in hepatitis C virus genotype 3 among Pakistani population. Ann Saudi Med. 2013;33(6):555–8.

21. Anagnostou O, Manolakopoulos S, Bakoyannis G, et al. Similar efficacy of antiviral therapy in chronic hepatitis C (CHC) patients with genotype 1 (G1) or genotype 4 (G4). Results of a large nationwide cohort study. Gastroenterology. 2011;140(5):S949.

22. Andre-Garnier E, Ribeyrol O, Gournay J, et al. Emergence of HCV resistance-associated variants in patients failing sofosbuvir-based regimens: an observational cohort. Antivir Ther. 2016.

23. Araujo ES, Dahari H, Neumann AU, et al. Very early prediction of response to HCV treatment with PEG-IFN-alfa-2a and ribavirin in HIV/HCV-coinfected patients. J Viral Hepat. 2011;18(4):e52–60.

24. Aronsohn A, Ancuta I, Caruntu F, et al. Impact of age on viral kinetics of peginterferon alfa-2a/ribavirin in chronic hepatitis C: Final analysis from the PROPHESYS cohort. J Viral Hepat. 2014;21(5):377–80.

25. Aronsohn A, Ancuta I, Florin, et al. Impact of age on virological response and relapse in patients with chronic hepatitis C (CHC) treated with peginterferon alfa-2a (40KD) (PegIFNa2a) plus ribavirin (RBV): Final results from the PROPHESYS multinational non-interventional cohort study. Hepatol Int. 2012;6(1):171–2.

26. Ascione A, Bruno S, Coppola C, et al. Hepatitis C treatment outcomes and predictors of response in treatment-naive patients treated with peginterferon alfa/ribavirin in real-world Italian clinics compared with other countries: Prophesys sub-analysis. J Hepatol. 2013;58:S322.

27. Ascione A, Bruno S, Coppola C, et al. Treatment outcomes and predictors of response in treatment-naive hepatitis C patients treated with Peg-interferon alpha/ribavirin in real-world Italian clinics: PROPHESYS sub-analysis. Dig Liver Dis. 2013;45:S34.

28. Ascione A, Carosi G, Mangia A, et al. Rates and predictors of sustained virological response and relapse in Italian naive mono-infected chronic hepatitis C patients treated with peginterferon alfa/ribavirin: Prophesys 2 final analysis. J Hepatol. 2012;56:S427–8.

29. Ashtari S, Vahedi M, Amin Pourhoseingholi M, et al. Evaluated outcomes in patients with chronic hepatitis C. Gastroenterol Hepatol Bed Bench. 2013;6(Suppl 1):S58–64.

30. Aziz H, Gil ML, Waheed Y, et al. Evaluation of prognostic factors for Peg Interferon alfa-2b plus ribavirin treatment on HCV infected patients in Pakistan. Infect Genet Evol. 2011;11(3):640–5.

31. Aziz H, Gill U, Raza A, et al. Metabolic syndrome is associated with poor treatment response to antiviral therapy in chronic hepatitis C genotype 3 patients. Eur J Gastroenterol Hepatol. 2014;26(5):538–43.

32. Aziz H, Khalid A, Murtaza S, et al. Treatment outcome in patients with Hepatitis C: Significance of baseline parameters and viral dynamics. Int J Infect Dis. 2011;15:S82.

33. Aziz H, Raza A, Ali K, et al. Polymorphism of the IL28B gene (rs8099917, rs12979860) and virological response of Pakistani hepatitis C virus genotype 3 patients to pegylated interferon therapy. Int J Infect Dis. 2015;30:91–7.

34. Aziz H, Raza A, Gill U, et al. Role of IL28B (rs8099917) polymorphism for SVR in HCV genotype 3 patients. Hepatol Int. 2014;8(1):S170.

35. Aziz H, Raza A, Irfan J. Optimum predictors of therapeutic outcome in HCV patients in Pakistan. J Med Virol. 2016;88(1):100–8.

36. Aziz H, Raza A, Waheed Y, et al. Analysis of variables and interactions among variables associated with a sustained virological response to pegylated interferon alfa-2a plus ribavirin in hepatitis C virus genotype 3-infected patients. Int J Infect Dis. 2012;16(8):e597–602.

37. Barcaui HS, Tavares GC, May SB, et al. Low rates of sustained virologic response with peginterferon plus ribavirin for chronic hepatitis C virus infection in HIV infected patients in Rio de Janeiro, Brazil. PLoS One. 2013;8(7):e67734.

38. Behnava B, Sharafi H, Keshvari M, et al. The role of polymorphisms near the IL28B gene on response to peg-interferon and ribavirin in thalassemic patients with hepatitis C. Hepat Mon. 2016;16(1):e32703.

39. Benjamin Y, Oo AM, Mei LY, et al. Predictors of treatment success in hepatitis C patients in a tertiary hospital in Singapore. Hepatol Int. 2012;6(1):167.

40. Bourke NM, O'Neill MT, Sarwar S, et al. In vitro blood cell responsiveness to IFN-α predicts clinical response independently of IL28B in hepatitis C virus genotype 1 infected patients. J Transl Med. 2014;12(1):206.

41. Bourlière M, Ouzan D, Rosenheim M, et al. Pegylated interferon-α2a plus ribavirin for chronic hepatitis C in a real-life setting: The Hepatys French cohort (2003-2007). Antivir Ther. 2012;17(1):101–10.

42. Brahmbhatt R, Rudloff R, McCashland T. DAA-containing regimens for the treatment of recurrent HCV infection after liver transplantation are safe and effective regardless of genotype and fibrosis stage. Gastroenterology. 2015;148(4):S1053.

43. Braniff C, Connelly C, Mollison L. Telemedicine allows successful Hepatitis C treatment in Western Australian prisons. J Viral Hepat. 2015;22:90.

44. Brjalin V, Salupere R, Tallo T, et al. Efficacy of peginterferon alpha-2A and ribavirin combination therapy in treatment-naive Estonian patients with chronic hepatitis C. Cent Eur J Public Health. 2012;20(2):150–5.

45. Brown R, Fried M, Reddy RK, et al. Daclatasvir and sofosbuvir therapy for patients with decompensated cirrhosis or post-liver transplant hcv recurrence and advanced fibrosis or cirrhosis: united states multicenter treatment protocol. J Hepatol. 2016(S814).

46. Brunner N, Senn O, Rosemann T, et al. Hepatitis C treatment for multimorbid patients with substance use disorder in a primary care-based integrated treatment centre: a retrospective analysis. Eur J Gastroenterol Hepatol. 2013;25(11):1300–7.

47. Bruno R, Cariti G, Nasta P, et al. OPERA: responses to peginterferon and ribavirin therapy in a subgroup of interferon-naive patients with HIV/HCV genotype 2/3 co-infection in Italy. Liver Int. 2015;35(1):120–9.

48. Bura M, Kowala-Piaskowska A, Adamek A, et al. Results of antiviral treatment of patients with chronic hepatitis C: experience of Poznan centre. Postepy Hig Med Dosw (Online). 2012;66:339–47.

49. Butt AS, Mumtaz K, Hamid S, et al. Retreatment of chronic hepatitis C, genotype 3 patients with pegylatedinterferon and ribavirin. Hepatol Int. 2011;5(1):247.

50. Cachay ER, Wyles D, Hill L, et al. The impact of direct-acting antivirals in the hepatitis C-sustained viral response in human immunodeficiency virus-infected patients with ongoing barriers to care. Open Forum Infect Dis. 2015;2(4):ofv168.

51. Campos-Varela I, Lai JC, Verna EC, et al. Hepatitis C genotype influences post-liver transplant outcomes. Transplantation. 2015;99(4):835–40.

52. Cardoso H, Albuquerque A, Moura MC, et al. The impact of cirrhosis in genotype 3 hepatitis C treatment outcome. Gastroenterology. 2014;146(5):S–259.

53. Carlin AF, Aristizabal P, Song Q, et al. Temporal dynamics of inflammatory cytokines/chemokines during sofosbuvir and ribavirin therapy for genotype 2 and 3 hepatitis C infection. Hepatology. 2015;62(4):1047–58.

54. Carosi G, Bruno R, Cariti G, et al. OPERA: use of pegylated interferon plus ribavirin for treating HCV-HIV coinfection in interferon-naive patients. Antivir Ther. 2014;19(8):735–45.

55. Castells L, Rimola A, Manzardo C, et al. Pegylated interferon plus ribavirin in HIV-infected patients with recurrent hepatitis C after liver transplantation: A prospective cohort study. J Hepatol. 2015;62(1):92–100.

56. Cha RR, Lee SS, Lee CM, et al. Clinical features and outcomes of patients with genotype 3 hepatitis C virus infection in Korea: A retrospective observational study. Medicine (Baltimore). 2016;95(6):e2755.

57. Charlton M, O'Leary J, Bzowej N, et al. Sofosbuvir/velpatasvir for the treatment of HCV decompensated liver disease patients: ASTRAL-4 study. Hepatol Int. 2016;10(1):S2.

58. Charlton M, Samuel D, Gane E, et al. Sofosbuvir and ribavirin for the treatment of recurrent hepatitis c infection after liver transplantation: Results of a prospective, multicenter study. Liver Transpl. 2014;20:S125.

59. Chaudhry A, Alam A, Nawaz A, et al. RVR is a strong predictor of SVR in HCV genotype 3 patients treated with standard interferon α2B and ribavirin. Am J Gastroenterol. 2012;107:S167–8.

60. Chaudhry A, Nawaz A, Riaz M, et al. Anemia as a predictor of SVR during treatment of chronic hepatitis C in patients undergoing treatment with interferon and ribavirin. Am J Gastroenterol. 2012;107:S168.

61. Cheetham TC, Niu F, Chiang K, et al. Factors associated with failure to achieve SVR in hepatitis C genotype 3 patients within an integrated care delivery system. J Manag Care Spec Pharm. 2015;21(8):641–7.

62. Cheetham TC, Tartof SY, Bider-Canfield Z, et al. Utilization and effectiveness of sofosbuvir based HCV regimens in a community based setting. Hepatology. 2015;62:769A–70A.

63. Chinnaswamy S, Das K, Bairagya BB, et al. Association of IL28B single nucleotide polymorphism rs8099917 with response to treatment in genotype 3 HCV-infected patients from India. Trop Gastroenterol. 2014;35(2):96–102.

64. Cho SH, Kim HJ, Han JY. Peginterferon and riabvirin treatment of virologic response rates for HCV infection in South Korea. Hepatol Int. 2013;7:S446–7.

65. Choudhary NS, Saigal S, Saraf N, et al. Genotype 3 and favorable IL28B polymorphism as predictors of good response for hepatitis C recurrence post liver transplantation. Liver Transpl. 2013;19(6):S146–7.

66. Christensen PB, Krarup HB, Laursen AL, et al. Negative HCV-RNA 2 weeks after initiation of treatment predicts sustained virological response to pegylated interferon alfa-2a and ribavirin in patients with chronic hepatitis C. Scand J Gastroenterol. 2012;47(8-9):1115–9.

67. Christensen S, Mauss S, Hueppe D, et al. Directly acting agents against HCV results from the German Hepatitis C Cohort (GECCO). CROI. 2016:233.

68. Chulanov V, Zhdanov K, Kersey K, et al. Sofosbuvir plus ribavirin for the treatment of russian patients with chronic HCV genotype 1 or 3 infection. Hepatology. 2014;60:676A–7A.

69. Clausen LN, Weis N, Ladelund S, et al. Genetic variants in the apoptosis gene BCL2L1 improve response to interferon-based treatment of hepatitis C virus genotype 3 infection. Int J Mol Sci. 2015;16(2):3213–25.

70. Coilly A, Fougerou C, De Ledinghen V, et al. The association of sofosbuvir and daclatasvir for treating severe recurrence of HCV infection after liver transplantation: Results from a large french prospective multicentric ANRS CO23 cupilt cohort. J Hepatol. 2015;62:S236–S7.

71. Collison M, Chin JL, Abu Shanab A, et al. Homozygosity for HLA group 2 alleles predicts treatment failure with interferon-alpha and ribavirin in chronic hepatitis C virus genotype 1 infection. J Interferon Cytokine Res. 2015;35(2):126–33.

72. Cornberg M, Buggisch P, Schober A, et al. treatment of patients with hepatitis C virus (HCV) genotype 3 infection in the era of direct acting antivirals (DAA): data from the german hepatitis C-registry (DHC-R). J Hepatol. 2016;64:S794.

73. Craxi A, Piccinino F, Ciancio A, et al. Real-world outcomes in patients with chronic hepatitis C: primary results of the PROBE study. Eur J Gastroenterol Hepatol. 2014;26(4):388–95.

74. Curry MP, O'Leary JG, Bzowej N, et al. Sofosbuvir and velpatasvir for HCV in patients with decompensated cirrhosis. N Engl J Med. 2015;373(27):2618–28.

75. Dalgard O, Martinot-Peignoux M, Verbaan H, et al. The usefulness of defining rapid virological response by a very sensitive assay (TMA) during treatment of HCV genotype 2/3 infection. PLoS One. 2015;10(8):e0120866.

76. Dalgard O, Weiland O, Noraberg G, et al. The effect of sofosbuvir containing regimes in patients with HCV genotype 3 infection - a Scandinavian real-life experience. J Hepatol. 2016;64:S803.

77. D'Ambrosio R, Poggiali E, Mazzoleni M, et al. Peg-interferon and ribavirin therapy is safe and effective among HCV patients with thalassaemia major. Dig Liv Dis. 2014;46:e19.

78. Deborah Friedman N, Green JH, Weber HM, et al. Hepatitis C virus treatment in the 'real-world': how well do 'real' patients respond? J Clin Exp Hepatol. 2014;4(3):214–20.

79. Delgado JS, Baumfeld Y, Novack V, et al. Efficacy of combined pegylated interferon and ribavirin therapy in Jewish patients of Israel suffering from chronic hepatitis C. Hepatol Int. 2011;5(4):985–90.

80. Deterding K, Buggisch P, Klinker H, et al. Safety and efficacy of IFN-free antiviral therapies in advanced HCV-associated liver cirrhosis: results from the German Hepatitis C-Registry (DHC-R). J Hepatol. 2016;64:S787.

81. Deterding K, Honer Zu Siederdissen C, Port K, et al. Improvement of liver function parameters in advanced HCV-associated liver cirrhosis by IFN-free antiviral therapies. Aliment Pharmacol Ther. 2015;42(7):889–901.

82. Deterding K, Höner Zu Siederdissen C, Port K, et al. Improvement of liver function parameters in advanced HCV-associated liver cirrhosis during IFN-free antiviral therapy. J Viral Hepat. 2015;22:92.

83. Di Marco V, Covolo L, Calvaruso V, et al. Who is more likely to respond to dual treatment with pegylated-interferon and ribavirin for chronic hepatitis C? A gender-oriented analysis. J Viral Hepat. 2013;20(11):790–800.

84. Di Marco V, Covolo L, Levrero M, et al. A multicenter prospective observational study to evaluate factors influencing efficacy, tolerance and compliance to antiviral tretament with interferon and ribavirin in daily clinical practice. Dig Liv Dis. 2011;43:S106.

85. Di Marco V, D'Ambrosio R, Bronte F, et al. Dual therapy with peg-interferon and ribavirin in thalassemia major patients with chronic HCV infection: Is there still an indication? Dig Liver Dis. 2016;48(6):650–5.

86. Dieterich D, Naggie S, Lalezari J, et al. Sofosbuvir plus ribavirin for HCV genotype 1-3 infection in HIV co-infected patients. Hepatol Int. 2014;8(1):S218–9.

87. Doehle B, Gontcharova V, Chodavarapu RK, et al. Resistance analysis of treatment-naïve HCV genotype 1-6 infected patients treated with sofosbuvir in combination with GS-5816 for 12 weeks. Hepatology. 2014;60:1138A–9A.

88. Dore GJ, Lawitz E, Hézode C, et al. Daclatasvir combined with peginterferon alfa-2a and ribavirin for 12 or 16 weeks in patients with hepatitis C virus genotype 2 or 3 infection: COMMAND GT2/3 study. J Gastroenterol Hepatol. 2013;28:155–6.

89. Dore GJ, Lawitz E, Hézode C, et al. Daclatasvir plus peginterferon and ribavirin is noninferior to peginterferon and ribavirin alone, and reduces the duration of treatment for HCV genotype 2 or 3 infection. Gastroenterology. 2015;148(2):355–66.

90. Eslam M, Aparcero R, Mousa YI, et al. Insulin resistance impairs viral dynamics independently of ethnicity or genotypes. J Clin Gastroenterol. 2012;46(3):228-34.

91. Eslam M, Leung R, Romero-Gomez M, et al. IFNL3 polymorphisms predict response to therapy in chronic hepatitis C genotype 2/3 infection. J Hepatol. 2014;61(2):235–41.

92. Esteban R, Nyberg L, Lalezari J, et al. Successful retreatment with sofosbuvir-containing regimens for HCV genotype 2 or 3 infected patients who failed prior sofosbuvir plus ribavirin therapy. J Hepatol. 2014;60(1):S4–5.

93. Evans A, Linzey D, Booth J. Ethnicity has no impact on SVR rates in patients with HCV genotype 3 treated with pegylated interferon and ribavirin. Gut. 2012;61:A131–2.

94. Everson GT, Towner WJ, Davis MN, et al. Sofosbuvir with velpatasvir in treatment-naive noncirrhotic patients with genotype 1 to 6 hepatitis c virus infection. Ann Intern Med. 2015;163(11):818–26.

95. Everson GT, Tran TT, Towner WJ, et al. Safety and efficacy of treatment with the interferon-free, ribavirin-free combination of sofosbuvir + GS-5816 for 12 weeks in treatment naive patients with genotype 1-6 HCV infection. J Hepatol. 2014;60(1):S46.

96. Faisal N, Mumtaz K, Marquez M, et al. High sustained virological response to pegylated interferon and ribavirin for recurrent genotype 3 hepatitis C infection post-liver transplantation. Hepatol Int. 2015;9(1):76–83.

97. Faisal N, Mumtaz K, Renner EL, et al. SVR rates of 71% following treatment with pegylated interferon and ribavirin for recurrent genotype 3 hepatitis c post liver transplantation: Retrospective analysis of a western single center series. Hepatology. 2013;58(4):1044A–5A.

98. Farley J, Truong A, Nguyen T, et al. Hepatitis C treatment outcome with pegylated interferon alpha 2a/ribavirin in a real life Community-based setting. Int J Infect Dis. 2012;16:e81–2.

99. Fateh A, Aghasadeghi MR, Keyvani H, et al. High resolution melting curve assay for detecting rs12979860 IL28B polymorphisms involved in response of Iranian patients to chronic hepatitis C treatment. Asian Pac J Cancer Prev. 2015;16(5):1873–80.

100. Fattovich G, Baroni GS, Pasino M, et al. Post-load insulin resistance does not predict virological response to treatment of chronic hepatitis C patients without the metabolic syndrome. Dig Liver Dis. 2012;44(5):419–25.

101. Fattovich G, Covolo L, Bibert S, et al. IL28B polymorphisms, IP-10 and viral load predict virological response to therapy in chronic hepatitis C. Aliment Pharmacol Ther. 2011;33(10):1162–72.

102. Feld J, Ramji A, Shafran S, et al. Ledipasvir/sofosbuvir with ribavirin for 12 weeks is effective and safe in treatment-naïve genotype-3 hepatitis C-infected patients in Canada. J Hepatol. 2016;64:S781.

103. Feld JJ, Nelson DR, Kowdley KV, et al. All oral therapy with sofosbuvir + ribavirin for 12 or 16 weeks in treatment experienced genotype 2/3 HCV-infected patients: The FUSION trial. Hepatol Int. 2013;7:S436.

104. Fernandez Rodriguez CM, Masnou H, Morillas R, et al. High vs standard dose of ribavirin plus peginterferonalfa- 2A in chronic hepatitis C, genotype 3 and high viral load. Final results of the dargen-3 study. J Hepatol. 2012;56:S437.

105. Fernández-Rodríguez A, Berenguer J, Rallón N, et al. PPARγ2 Pro12Ala polymorphism is associated with sustained virological response in HIV/HCV-coinfected patients under HCV therapy. J Acquir Immune Defic Syndr. 2014;67(2):113–9.

106. Fernández-Rodríguez CM, Morillas RM, Masnou H, et al. Randomized clinical trial comparing high versus standard dose of ribavirin plus peginterferon alfa-2a in hepatitis C genotype 3 and high viral load. Dargen-3 study. Gastroenterol Hepatol. 2014;37(1):1–8.

107. Firdaus R, Biswas A, Saha K, et al. Impact of host IL28B rs12979860, rs8099917 in interferon responsiveness and advanced liver disease in chronic genotype 3 hepatitis C patients. PLoS One. 2014;9(6):e99126.

108. Fontaine H, Lacombe K, Dhiver C, et al. Daclatasvir plus sofosbuvir with or without ribavirin in patients with hiv-hcv co-infection: Interim analysis of a french multicenter compassionate use program. J Hepatol. 2015;62:S275.

109. Foster G, Chayama K, Chuang WL, et al. Efficacy and safety of peginterferon lambda 1a with or without daclatasvir compared with peginterferon alfa 2A each in combination with ribavirin in treatment naive patients with chronic hepatitis C virus genotype 2 or 3 infection. Hepatol Int. 2015;9(1):S259.

110. Foster G, Shoeb D, Weatherall A, et al. Randomised controlled trial of 24 and 48 weeks of pegylated interferon alfa 2a plus ribavirin in patients with genotype 3 HCV and cirrhosis. J Hepatol. 2013;58:S335.

111. Foster GR, Afdhal N, Roberts SK, et al. Sofosbuvir and Velpatasvir for HCV Genotype 2 and 3 Infection. N Engl J Med. 2015;373(27):2608–17.

112. Foster GR, Coppola C, Derbala M, et al. Impact of safety-related dose reductions or discontinuations on sustained virologic response in HCV-infected patients: results from the GUARD-C cohort. PLoS One. 2016;11(3):e0151703.

113. Foster GR, Hezode C, Bronowicki JP, et al. Telaprevir alone or with peginterferon and ribavirin reduces HCV RNA in patients with chronic genotype 2 but not genotype 3 infections. Gastroenterology. 2011;141(3):881–9.

114. Foster GR, Irving WL, Cheung MC, et al. Impact of direct acting antiviral therapy in patients with chronic hepatitis C and decompensated cirrhosis. J Hepatol. 2016;64:1224–31.

115. Foster GR, McLauchlan J, Irving W, et al. Treatment of decompensated HCV cirrhosis in patients with diverse genotypes: 12 weeks sofosbuvir and ns5a inhibitors with/without ribavirin is effective in HCV genotypes 1 and 3. J Hepatol. 2015;62:S190–1.

116. Foster GR, Pianko S, Brown A, et al. Efficacy of sofosbuvir plus ribavirin with or without peginterferon-alfa in patients with hepatitis C virus genotype 3 infection and treatment-experienced patients with cirrhosis and hepatitis C virus genotype 2 infection. Gastroenterology. 2015;149(6):1462–70.

117. Foster GR, Pianko S, Cooper C, et al. Sofosbuvir + peginterferon/ribavirin for 12 weeks vs sofosbuvir + ribavirin for 16 or 24 weeks in genotype 3 HCV infected patients and treatment-experienced cirrhotic patients with genotype 2 HCV: The BOSON study. J Hepatol. 2015;62:S259–60.

118. Fougerou-Leurent C, Coilly A, Veislinger A, et al. The association of sofosbuvir and daclatasvir for treating severe recurrence of HVC infection after liver transplantation: Results from the large French prospective multicentric ANRS CO23 CUPILT cohort. Fundam Clin Pharmacol. 2015;29:43.

119. Francioso S, Almerighi C, Forte P, et al. A simple rule to personalize standard dual therapy across all genotypes in naive chronic hepatitis C patients: the TT4 randomized trial. Dig Liver Dis. 2014;46(2):164–9.

120. Fried MW, Hadziyannis SJ, Shiffman ML, et al. Rapid virological response is the most important predictor of sustained virological response across genotypes in patients with chronic hepatitis C virus infection. J Hepatol. 2011;55(1):69–75.

121. Gane E, Hyland RH, Pang P, et al. Sofosbuvir/ledipasvir fixed dose combination is safe and effective in HCV infected populations including decompensated patients and patients with prior sofosbuvir treatment experience. Gastroenterology. 2014;146(5):S904–5.

122. Gane E, Lawitz E, Rodriguez-Torres M, et al. Phase 3 randomized controlled trial of all-oral treatment with sofosbuvir + ribavirin for 12 weeks compared to 24 weeks of PEG + ribavirin in treatment-naïve GT2/3 HCV-infected patients (fission). J Hepatol. 2013;58:S3.

123. Gane E, Nahass R, Luketic V, et al. Efficacy of 12 or 18 weeks of grazoprevir plus elbasvir with ribavirin in treatment-naive, noncirrhotic HCV genotype 3-infected patients. J Hepatol. 2015;62:S621.

124. Gane E, Stedman CAM, Asselah T, et al. Ledipasvir/sofosbuvir with or without ribavirin for the treatment of patients with genotype 2-6 chronic HCV infection: Summary results from four phase ii studies. Am J Gastroenterol. 2015;110:S870–1.

125. Gane E, Svarovskaia ES, Dvory-Sobol H, et al. Resistance analysis of genotype 1 or 3 HCV-infected patients treated with sofosbuvir/velpatasvir+GS-9857 for 6 or 8 weeks. J Hepatol. 2016;64:S401–2.

126. Gane EJ, Hyland RH, An D, et al. Once daily sofosbuvir with GS-5816 for 8 weeks with or without ribavirin in patients with HCV genotype 3 without cirrhosis result in high rates of SVR12: The electron2 study. Hepatology. 2014;60:236A–7A.

127. Gane EJ, Hyland RH, An D, et al. Sofosbuvir/ledipasvir fixed dose combination is safe and effective in difficult-to-treat populations including genotype-3 patients, decompensated genotype-1 patients, and genotype-1 patients with prior sofosbuvir treatment experience. J Hepatol. 2014;60(1):S3–4.

128. Gane EJ, Hyland RH, An D, et al. Efficacy of ledipasvir and sofosbuvir, with or without ribavirin, for 12 weeks in patients with HCV genotype 3 or 6 infection. Gastroenterology. 2015;149(6):1454–61.

129. Gane EJ, Hyland RH, An D, et al. High efficacy of LDV/SOF regimens for 12 weeks for patients with HCV genotype 3 or 6 infection. Hepatology. 2014;60(6):1274A–5A.

130. Gane EJ, Hyland RH, Yang Y, et al. Sofosbuvir/GS-5816+GS-9857 for 6 or 8 weeks in genotype 1 or 3 HCV-infected patients. Hepatology. 2015;62:226A.

131. Gane EJ, Lawitz E, Mangia A, et al. Sofosbuvir in treatment-naïve patients with hepatitis C infection. J Gastroenterol Hepatol. 2013;28:165–6.

132. Gane EJ, Lawitz E, Rodriguez-Torres M, et al. Phase 3 randomized controlled trial comparing sofosbuvir + ribavirin for 12 weeks to PEG + ribavirin in treatment-naïve GT2/3 HCV-infected patients: the FISSION trial. Hepatol Int. 2013;7:S435–S6.

133. Gane EJ, Nguyen M, Kwo P, et al. Short duration treatment with sofosbuvir/velpatasvir plus GS-9857 in treatment-naive genotype 1–6 HCV infected patients with or without cirrhosis. J Hepatol. 2016;64:S758–9.

134. Gane EJ, Pianko S, Roberts SK, et al. High efficacy of an 8-week 3-drug regimen of grazoprevir/MK-8408/MK-3682 in HCV genotype 1, 2 and 3-infected patients: SVR24 data from the phase 2 C-CREST 1 and 2 studies. J Hepatol. 2016;64:S759.

135. Gane EJ, Pianko S, Roberts SK, et al. Phase 2, randomized, open-label clinical trials of the efficacy and safety of grazoprevir and MK-3682 (NS5B polymerase inhibitor) with either elbasvir or MK-8408 (NS5A inhibitor) in patients with chronic HCV GT1, 2 or 3 infection (Part A of C-CREST-1 & 2). Hepatology. 2015;62(6):1389A.

136. Gane EJ, Stedman CA, Hyland RH, et al. Electron: Once daily PSI-7977 plus RBV in HCV GT1/2/3. J Hepatol. 2012;56:S438–9.

137. George J, Hillson JL, Leggett B, et al. Peginterferon Lambda-1a (Lambda) compared to peginterferon alfa-2a (alfa) in treatment-naïve patients with HCV genotypes (G) 2 or 3: Fi rst SVR24 results from emerge phase IIb. J Gastroenterol Hepatol. 2012;27:165–6.

138. Georgie F, Nafisi S, Kohli A, et al. Primary care physicians utilizing the ECHO model equally effective as subspecialists treating HCV using direct-acting antivirals-only regimens: Results of the ECHO model. J Hepatol. 2016;64:S818–9.

139. Gill M, Niaz T, Khan S, et al. Outcomes of pegylated interferon alfa-2b plus ribavirin therapy in treatment naive HCV cirrhotic patients. Hepatol Int. 2014;8(1):S164.

140. Gill U, Aziz H, Gill ML. Rapid virological response tailors the duration of treatment in hepatitis C virus genotype 3 patients treated with pegylated interferon alfa-2a and ribavirin in Pakistan. Int J Infect Dis. 2013;17(11):e1017–21.

141. Grebely J, Dalgard O, Conway B, et al. Efficacy of response-guided pegylated interferon and ribavirin therapy for people who inject drugs with HCV genotype 2/3 infection: The activate study. J Hepatol. 2015;62:S657.

142. Gupta AC, Trehanpati N, Sukriti S, et al. Interleukin-28b CC genotype predicts early treatment response and CT/TT genotypes predicts non-response in patients infected with HCV genotype 3. J Med Virol. 2014;86(4):707–12.

143. Gupta V, Kumar A, Sharma P, et al. Sustained virological response rates to antiviral therapy in genotype 1 and 3 chronic hepatitis C patients: A study from North India. J Clin Exp Hepatol. 2014;4(4):287–92.

144. Gürbüz Y, Tülek NE, Tütüncü EE, et al. Evaluation of dual therapy in real life setting in treatment-naïve Turkish patients with HCV infection: A multicenter, retrospective study. Balkan Med J. 2016;33(1):18–26.

145. Hajiaghamohammadi A, Samimi R, Miroliaee A, et al. Treatment outcome in chronic hepatitis C infection: a four years survey among Iranian patients. Glob J Health Sci. 2015;7(3):75–81.

146. Haj-Sheykholeslami A, Keshvari M, Sharafi H, et al. Interferon-lambda polymorphisms and response to pegylated interferon in Iranian hepatitis C patients. World J Gastroenterol. 2015;21(29):8935–42.

147. Hansen N, Obel N, Christensen PB, et al. Effectiveness of treatment with pegylated interferon and ribavirin in an unselected population of patients with chronic hepatitis C: a Danish nationwide cohort study. BMC Infect Dis. 2011;11:177.

148. Hassanein T, Poordad F, Kwo P, et al. High SVR rates with ABT-493 + ABT-530 in non-cirrhotic patients with HCV genotypes 1, 2, 3 infection. Hepatol Int. 2016;10(1):S143.

149. Heidari M, Bayani M, Bijani A, et al. Factors influencing therapeutic response to pegylated interferon plus ribavirin in the different genotypes of chronic hepatitis C. Caspian J Intern Med. 2014;5(4):219–22.

150. Heidrich B, Cordes HJ, Klinker H, et al. Treatment extension of pegylated interferon alpha and ribavirin does not improve SVR in patients with genotypes 2/3 without rapid virological response (OPTEX trial): A prospective, randomized, two-arm, multicentre phase IV clinical trial. PLoS One. 2015;10(6):e0128069.

151. Heidrich B, Wiegand SB, Buggisch P, et al. Treatment of naive patients with chronic hepatitis C genotypes 2 and 3 with pegylated interferon alpha and ribavirin in a real world setting: relevance for the new era of DAA. PLoS One. 2014;9(10):e108751.

152. Hennessy T, Kumar R, Wigg A, et al. Hepatitis C & porphyria cutanea tarda experience in 2 tertiary centres. J Gastroenterol Hepatol. 2013;28:160.

153. Herzer K, Welzel TM, Ferenci P, et al. Daclatasvir in combination with sofosbuvir with or without ribavirin is safe and efficacious in liver transplant recipients with HCV recurrence: Interim results of a multicenter compassionate use program. Hepatology. 2015;62:341A.

154. Hezode C, De Ledinghen V, Fontaine H, et al. Daclatasvir plus sofosbuvir with or without ribavirin in patients with HCV genotype 3 infection: Interim analysis of a French multicenter compassionate use program. J Viral Hepat. 2015;22:29.

155. Hezode C, De Ledinghen V, Fontaine H, et al. Daclatasvir plus sofosbuvir with or without ribavirin in genotype 3 patients from a large French multicenter compassionate use program. Hepatology. 2015;62:314A.

156. Hezode C, Dore G, Pianko S, et al. Daclatasvir plus sofosbuvir plus ribavirin for 12 or 16 weeks in treatment-experienced patients with HCV genotype 3 infection and advanced fibrosis or cirrhosis. J Hepatol. 2016;64:S753–4.

157. Hezode C, Reau N, Svarovskaia E, et al. Resistance analysis in 1284 patients with genotype 1 to 6 HCV infection treated with sofosbuvir/velpatasvir in the phase 3 ASTRAL-1, ASTRAL-2, ASTRAL-3 and ASTRAL-4 studies. J Hepatol. 2016;64:S399–400.

158. Hezode C, Zeuzem S, Salupere R, et al. High sustained response rates with sofosbuvir + ribavirin for 12 or 24 weeks for patients with HCV genotype 2 or 3 infection: The valence trial. Hepatol Int. 2014;8(1):S220–1.

159. Hofmann WP, Mauss S, Lutz T, et al. Benefit of treatment individualization in patients with chronic hepatitis C receiving peginterferon alfa-2a and ribavirin in a large noninterventional cohort study. PLoS One. 2015;10(7):e0134839.

160. Hwang EW, Thomas IC, Backus LI. Pre-treatment predictors of rapid virologic response (RVR) and the predictive value of rvr on sustained virologic response in U.S. veterans with chronic hepatitis C virus infection. Gastroenterology. 2011;140(5):S910.

161. Hwang EW, Thomas IC, Cheung R, et al. Implications of rapid virological response in hepatitis C therapy in the US veteran population. Aliment Pharmacol Ther. 2012;35(1):105–15.

162. Ingiliz P, Schewe K, Lutz T, et al. Sofosbuvir-based treatments for patients with hepatitis C virus (HCV) mono-infection and human immunodeficiency virus (HIV)-HCV co-infection with genotype 3 in clinical practice - results from the GErman hepatitis C COhort (GECCO). Hepatology. 2015;62:729A–30A.

163. Inglot M, Pawlowski T, Szymczak A, et al. Replication of hepatitis C virus in peripheral blood mononuclear cells in patients with chronic hepatitis C treated with pegylated interferon alpha and ribavirin. Postepy Hig Med Dosw (Online). 2013;67:186–91.

164. Iqbal M, Zohair Nomani A, Saleem Qureshi M, et al. Treatment induced decline in hematological cell lines as a predictor of response to treatment with conventional interferon plus ribavirin for chronic hepatitis C infection in Pakistan. World J Med Sci. 2013;8(3):190–4.

165. Iqbal S, Khalil Ur R, Sheikh MA, et al. Response of different HCV genotypes to interferon therapy in different age groups of chronic hepatitis-C patients. J Ayub Med Coll Abbottabad. 2014;26(3):310–5.

166. Isaksen K, Aabakken L, Grimstad T, et al. Hepatitis C treatment at three Norwegian hospitals 2000-2011. Tidsskr Nor Laegeforen. 2015;135(22):2052–8.

167. Isaksen K, Aabakken L, Sandvei PK, et al. Age is a strong predictor of SVR in patients infected with hepatitis C genotype 3. Hepatology. 2013;58(4):1118A.

168. Ismail MH. Prediction of sustained virologic responses to combination therapy of pegylated interferon-α and ribavirin in patients with chronic hepatitis C infection. Journal of Family and Community Medicine. 2013;20(1):35–40.

169. Iwata R, Stieger B, Mertens JC, et al. The role of bile acid retention and a common polymorphism in the ABCB11 gene as host factors affecting antiviral treatment response in chronic hepatitis C. J Viral Hepat. 2011;18(11):768–78.

170. Jablonowska E, Piekarska A, Koslinska-Berkan E, et al. Sustained virologic response and IL28B single-nucleotide polymorphisms in patients with chronic hepatitis C treated with pegylated interferon alfa and ribavirin. Acta Biochim Pol. 2012;59(3):333–7.

171. Jacobson I, Yoshida EM, Sulkowski M, et al. Treatment with sofosbuvir + ribavirin for 12 weeks achieves SVR12 of 78% in GT2/3 interferon-ineligible, -intolerant, or -unwilling patients: Results of the phase 3 positron trial. J Hepatol. 2013;58:S28.

172. Jacobson IM, Gordon SC, Kowdley KV, et al. Sofosbuvir for hepatitis C genotype 2 or 3 in patients without treatment options. N Engl J Med. 2013;368(20):1867–77.

173. Jaideep S, Azmi AN, Rosaida MS. Outcomes of peg-interferon alpha use in treatment of hepatitis C in a tertiary gastroenterology hospital in Malaysia. Hepatol Int. 2014;8(1):S201.

174. James PD, Wong D. Optimizing hepatitis C therapy in HIV/hepatitis C virus (HCV) coinfected patients: Analysis of HCV viral kinetics on treatment. Can J Infect Dis Med Microbiol. 2012;23(1):31–5.

175. Jindal A, Sharma M, Goswami A, et al. Sofosbuvir and ribavirin for chronic hepatitis C: Interim analysis from a real-life Indian cohort. Hepatol Int. 2016;10(1):S151–2.

176. Jongboonyanuparp T, Witeerungrot T, Chamroonkul N, et al. Efficacy and tolerability of pegylated interferon plus ribavirin therapy in thai patients with chronic hepatitis C: Real world practice. Hepatol Int. 2011;5(1):242.

177. Kakkar S, Sobti PC, Markan A. Safety and efficacy of interferon and ribavarin therapy in HCV positive thalassemic patients. Haematologica. 2014;99:742.

178. Kakkar S, Sobti PC, Sood A, et al. Safety and efficacy of interferon and ribavirin therapy in thalassemia patients with chronic hepatitis c. Indian J Hematol Blood Transfus. 2013;29(4):366.

179. Khaitova V, Chernyak M, Motamed DB, et al. Escalating-dose pegylated interferon and ribavirin for treatment of recurrent hepatitis C in the post-transplant population: A singlecenter study. Hepatology. 2011;54:808A.

180. Khan AJ, Saraswat VA, Choudhuri G, et al. Association of interleukin-10 polymorphisms with chronic hepatitis C virus infection in a case-control study and its effect on the response to combined pegylated interferon/ribavirin therapy. Epidemiol Infect. 2015;143(1):71–80.

181. Kim HJ, Kim JH, Kim JI, et al. Peginterferon and riabvirin treatment of virologic response rates for HCV infection in South Korea. J Gastroenterol Hepatol. 2012;27:240–1.

182. Kleinstein SE, Urban TJ, Long N, et al. Genome-wide association study of response to sofosbuvir-ribavirin treatment in GT2/3 HCV patients. J Hepatol. 2015;62:S590.

183. Kotsiri I, Hadziyannis E, Georgiou A, et al. Changes in serum transforming growth factor-beta1 levels in chronic hepatitis C patients under antiviral therapy. Ann Gastroenterol. 2016;29(1):79–84.

184. Kowdley K, Angus P, Bernstein D, et al. All-oral treatment with daclatasvir plus sofosbuvir ± ribavirin in HCV genotype 3-infected patients with advanced fibrosis or cirrhosis: an analysis of ALLY-3 and ALLY-3+. J Hepatol. 2016;64:S789–90.

185. Kowdley K, Bacon B, Dieterich D, et al. Efficacy evaluation of 24 week SOF + RBV in a heterogeneous, real-world population of genotype 3 HCV patients; data from the trio network. J Hepatol. 2015;62:S665–S6.

186. Kowdley K, Nelson D, Lalezari J, et al. On-treatment HCV RNA as a predictor of sustained virologic response in HCV genotype 3-infected patients treated with daclatasvir and sofosbuvir: Analysis of phase 3 ALLY-3 study. Gastroenterology. 2015;148(4):S1084.

187. Kowdley K, Shiffman M, Sheikh A, et al. Sofosbuvir + ribavirin with or without peginterferon is well-tolerated and associated with high svr rates: Integrated results from 4 phase 3 trials in hcv genotype 1-6. Am J Gastroenterol. 2013;108:S123.

188. Kozielewicz D, Dybowska D, Halota W, et al. Natural leukocyte interferon alpha (Alfaferone) combined with ribavirin in the treatment of patients with HCV-related cirrhosis: our experience. Infection. 2011;39(5):433–7.

189. Kravchenko A, Kanestri V, Kuimova U, et al. Using phosphazide in regimes of ART in patients co-infected with HIV and HCV receiving treatment of hepatitis. J Int AIDS Soc. 2012;15:90.

190. Kurincic TS, Lesnicar G, Poljak M, et al. Impact of added fluvastatin to standard-of-care treatment on sustained virological response in Naïve chronic hepatitis C patients infected with genotypes 1 and 3. Intervirology. 2013;57(1):23–30.

191. Kwo PY, Bennett M, Wang S, et al. High SVR4 rates achieved with the next generation NS3/4A Protease inhibitor ABT-493 and NS5A inhibitor ABT-530 in non-cirrhotic treatment-naïve and treatment-experienced patients with HCV genotype 3 infection (SURVEYOR-2). Hepatology. 2015;62:337A–8A.

192. Kwo PY, Fried MW, Reddy KR, et al. Daclatasvir and sofosbuvir in patients with recurrent HCV Following liver transplantation and advanced fibrosis or cirrhosis: United States multicenter treatment protocol. Hepatology. 2015;62:321A.

193. Kwo PY, Wyles DL, Wang S, et al. 100% SVR4 with ABT-493 and ABT-530 with or without ribavirin in treatment-naïve HCV genotype 3-infected patients with cirrhosis. J Hepatol. 2016;64:S208.

194. Lacombe K, Fontaine H, Dhiver C, et al. Daclatasvir plus sofosbuvir with or without ribavirin in patients with HIV-HCV co-infection: Interim analysis of a French multicentre compassionate use programme. J Int AIDS Soc. 2015;18:42–3.

195. Lagging M, Rembeck K, Rauning Buhl M, et al. Retreatment with peg-interferon and ribavirin in patients with chronic hepatitis C virus genotype 2 or 3 infection with prior relapse. Scand J Gastroenterol. 2013;48(7):839–47.

196. Lange CM, Bibert S, Kutalik Z, et al. A genetic validation study reveals a role of vitamin D metabolism in the response to interferon-alfa-based therapy of chronic hepatitis C. PLoS One. 2012;7(7):e40159.

197. Lange CM, Kutalik Z, Morikawa K, et al. Serum ferritin levels are associated with a distinct phenotype of chronic hepatitis C poorly responding to pegylated interferon-alpha and ribavirin therapy. Hepatology. 2012;55(4):1038–47.

198. Latt NL, Louie V, Gharibian D, et al. Treatment of hepatitis C infection: Single-center, real world experience with sofosbuvir-based therapies. Am J Gastroenterol. 2015;110:S914–5.

199. Lawitz E, Kowdley K, Curry M, et al. High efficacy of sofosbuvir/velpatasvir plus GS-9857 for 12 weeks in treatment-experienced genotype 1-6 HCV infected patients, including those previously treated with direct-acting antivirals. J Hepatol. 2016;64:S139–40.

200. Lawitz E, Mangia A, Wyles D, et al. Sofosbuvir for previously untreated chronic hepatitis C infection. N Engl J Med. 2013;368(20):1878-87.

201. Lawitz E, Poordad F, Brainard DM, et al. Sofosbuvir with peginterferon-ribavirin for 12 weeks in previously treated patients with hepatitis C genotype 2 or 3 and cirrhosis. Hepatology. 2015;61(3):769–75.

202. Lawitz E, Poordad FF, Brainard D, et al. Sofosbuvir in combination with PegIFN and ribavirin for 12 weeks provides high SVR rates in HCV-infected genotype 2 or 3 treatment experienced patients with and without compensated cirrhosis: Results from the LONESTAR-2 study. Hepatol Int. 2014;8(1):S219–20.

203. Lawitz E, Sullivan G, Rodriguez-Torres M, et al. Exploratory trial of ombitasvir and ABT-450/r with or without ribavirin for HCV genotype 1, 2, and 3 infection. J Infect. 2015;70(2):197–205.

204. Lawitz E, Sullivan G, Rodriguez-Torres M, et al. A 12-week trial of interferon-free regimens containing ABT-450/r and ABT-267 +/-ribavirin (RBV) in treatment-naïve patients with HCV genotypes 1-3. Hepatol Int. 2013;7:S358–9.

205. Lawitz E, Zeuzem S, Stedman CA, et al. Sofosbuvir-based regimens for patients with hepatitis C virus genotype 3 infection: Summary results from the valence, lonestar-2, and electron-2 studies. Gastroenterology. 2015;148(4):S1085–6.

206. Lawson A, Irving W. A comparison of the natural history and outcome of treatment for Asian and non-Asian hepatitis C-infected patients. J Viral Hepat. 2011;18(7):e270-7.

207. Leise M, Rodriguez B, Pungpapong S, et al. Effectiveness of sofosbuvir and ribavirin for genotype 2 and 3 HCV among non-cirrhotic, cirrhotic, and post-transplant patients at 3 us medical centers. J Hepatol. 2015;62:S659.

208. Leroy V, Angus P, Bronowicki JP, et al. Daclatasvir + sofosbuvir + ribavirin for HCV GT-3 with cirrhosis or advanced fibrosis: ALLY-3 + study. Hepatol Int. 2016;10(1):S19.

209. Leroy V, Angus PW, Bronowicki J, et al. All-oral treatment with daclatasvir (DCV) plus sofosbuvir (SOF) plus ribavirin (RBV) for 12 or 16 weeks in HCV genotype (GT) 3-infected patients with advanced fibrosis or cirrhosis: the ALLY-3+ phase 3 study. Hepatology. 2015:LB-3.

210. Lewis DJ, Kalra B, Chiu WL, et al. Thyroid dysfunction in patients treated with interferon & ribavirin for hepatitis C virus infection. J Gastroenterol Hepatol. 2013;28:58–9.

211. Lim KBL, Sima HR, Fiel MI, et al. Utility of the low-accelerating-dose regimen in 182 liver recipients with recurrent hepatitis C virus. World J Gastroenterol. 2015;21(20):6236–45.

212. Lin JA, Chen YC, Cheng SN, et al. Peginterferon alfa-2a plus ribavirin for hemophilic patients with chronic hepatitis C virus infection in Taiwan. J Formos Med Assoc. 2014;113(10):727–33.

213. Lin ON, Nguyen N, Chang CY, et al. Safety and effectiveness of sofosbuvir (SOF) combined with ribavirin (RBV) for the treatment of genotype 2 and 3 chronic hepatitis C infection (HCV-2 and HCV-3). Gastroenterology. 2015;148(4):S1092.

214. Livingston SE, Townshend-Bulson LJ, Bruden DJ, et al. Results of interferon-based treatments in Alaska Native and American Indian population with chronic hepatitis C. Int J Circumpolar Health. 2016;75:30696.

215. Lopez-Cortes LF, Ruiz-Valderas R, Jimenez-Jimenez L, et al. Influence of IL28B polymorphisms on response to a lower-than-standard dose peg-IFN-alpha 2a for genotype 3 chronic hepatitis C in HIV-coinfected patients. PLoS One. 2012;7(1):e28115.

216. Luetkemeyer A, Sulkowski M, Naggie S, et al. Sofosbuvir plus ribavirin for HCV genotype 1-3 infection in HIV co-infected patients (PHOTON-1). Can J Infect Dis Med Microbiol. 2014;25:18A–9A.

217. Luetkemeyer AF, McDonald C, Ramgopal M, et al. 12 weeks of daclatasvir in combination with sofosbuvir for HIV-HCV Coinfection (ALLY-2 Study): Efficacy and safety by HIV combination antiretroviral regimens. Clin Infect Dis. 2016.

218. Maasoumy B, Vermehren J, Welker MW, et al. Clinical value of on-treatment HCV RNA levels during different approved sofosbuvir-based antiviral regimens. J Hepatol. 2016.

219. Maieron A, Metz-Gercek S, Scherzer TM, et al. Shortening of treatment duration in patients with chronic hepatitis C genotype 2 and 3 - impact of ribavirin dose - a randomized multicentre trial. BMC Res Notes. 2011;4:220.

220. Mandorfer M, Schwabl P, Steiner S, et al. Interferon-free treatment with sofosbuvir/daclatasvir achieves sustained virologic response in 100% of HIV/hepatitis C virus-coinfected patients with advanced liver disease. Aids. 2016;30(7):1039–47.

221. Mangia A, Roberts SK, Pianko S, et al. Sofosbuvir/GS-5816 fixed dose combination for 12 weeks compared to sofosbuvir with ribavirin for 24 weeks in genotype 3 HCV infected patients: The randomized controlled phase 3 ASTRAL-3 Study. Hepatology. 2015;62:338A–9A.

222. Manns M, Zeuzem S, Sood A, et al. Reduced dose and duration of peginterferon alfa-2b and weight-based ribavirin in patients with genotype 2 and 3 chronic hepatitis C. J Hepatol. 2011;55(3):554–63.

223. Maraver M, Rojas L, Ampuero J, et al. Factors involved in sustained viral response (SVR) achievement in patients with favorable IL28B CC genotype in Hepatitis C. J Hepatol. 2012;56:S353–4.

224. Marcellin P, Cheinquer H, Curescu M, et al. High sustained virologic response rates in rapid virologic response patients in the large real-world PROPHESYS cohort confirm results from randomized clinical trials. Hepatology. 2012;56(6):2039–50.

225. Marciano S, Borzi SM, Dirchwolf M, et al. Pre-treatment prediction of response to peginterferon plus ribavirin in chronic hepatitis C genotype 3. World J Hepatol. 2015;7(4):703–9.

226. Maribel RT, Susanna N, Mark S, et al. Sofosbuvir plus ribavirin for HCV genotype 1-3 infection in HIV co-infected patients (PHOTON-1). J Int AIDS Soc. 2014;17:18–.

227. Marotta P, Bailey R, Elkashab M, et al. Real-life effectiveness of peginterferon alpha 2B plus ribavirin in a canadian cohort of treatment-naïve chronic hepatitis C patients with genotype 2 and genotype 3: Results from the power and redipen studies. Can J Infect Dis Med Microbiol. 2015;26:66B.

228. Mauss S, Hueppe D, Zehnter E, et al. Age- and gender-related differences in SVR and relapse following treatment of HCV genotype-3 (G3) infection with peginterferonalfa-2b/ribavirin(Peg2b/RBV) in real-life. Hepatol Int. 2013;7:S353–4.

229. Mauss S, Hueppe D, Zehnter E, et al. Age- and gender-related differences in svr and relapse following treatment of hcv genotype 3 (G3) Infection with Peginterferon Alfa-2b (Peg2b)/Ribavirin (RBV) in real-life. Hepatology. 2012;56:589A.

230. Mauss S, Teuber G, Zehnter E, et al. Treatment of HCV genotype 3 (GT3)-infection with Peg-IFNa-2b and ribavirin (RBV): Age related differences in SVR and relapse. J Hepatol. 2011;54:S177.

231. Mazzaro C, Monti G, Saccardo F, et al. Efficacy and safety of peginterferon alfa-2b plus ribavirin for HCV-positive mixed cryoglobulinemia: a multicentre open-label study. Clin Exp Rheumatol. 2011;29(6):933–41.

232. Michael C, Edward G, Michael P M, et al. Sofosbuvir and ribavirin for the treatment of established recurrent hepatitis C infection after liver transplantation: Preliminary results of a prospective, multicenter study. Hepatol Int. 2014;8(1):S224–S5.

233. Moghaddam A, Melum E, Reinton N, et al. IL28B genetic variation and treatment response in patients with hepatitis C virus genotype 3 infection. Hepatology. 2011;53(3):746–54.

234. Molina JM, Orkin C, Iser DM, et al. Sofosbuvir plus ribavirin for treatment of hepatitis C virus in patients co-infected with HIV (PHOTON-2): a multicentre, open-label, non-randomised, phase 3 study. Lancet. 2015;385(9973):1098–106.

235. Mollison L, Cheng W, Miczkova S, et al. Improved sustained virological response with pegylatedinterferon alpha-2b compared with alpha-2a, (and ribavrin) in chronic hepatitis C genotypes 2 and 3. Hepatol Int. 2013;7:S412.

236. Muir AJ, Arora S, Everson G, et al. A randomized phase 2b study of peginterferon lambda-1a for the treatment of chronic HCV infection. J Hepatol. 2014;61(6):1238–46.

237. Muir AJ, Strasser S, Wang S, et al. High SVR rates with ABT-493 + ABT-530 co-administered for 8 weeks in non-cirrhotic patients with HCV genotype 3 infection. J Hepatol. 2016;64(S186).

238. Mulkay JP, Bourgeois S, Lasser L, et al. Characteristics, treatment, and virologic responses of chronic hepatitis C patients treated with peginterferon alfa-2a and ribavirin in Belgium: A sub-analysis of the PROPHESYS study. Acta Gastroenterol Belg. 2014;77(1):30–40.

239. Muller K, Rodgers A, Wundke R, et al. Single centre experience with pegylated interferon and ribavirin for hepatitis C: looking back before moving forward. Intern Med J. 2012;42(7):765–72.

240. Mutalib N, Tan SS, Omar H, et al. The disease profiles and outcome of hepatitis C infection a single centre experience. Hepatol Int. 2015;9(1):S265.

241. Nafisi S, Fante G, Moore A, et al. Limited effectiveness of sofosbuvir and ribavirin for the treatment of HCV GT-3 in patients with cirrhosis: A real-world GT-3 HCV treatment experience. Hepatology. 2015;62:784A–5A.

242. Naggie S, Sulkowski M, Lalezari J, et al. Sofosbuvir plus ribavirin for HCV genotype 1-3 infection in hiv coinfected patients (PHOTON-1). Top Antivir Med. 2014;22(e-1):14.

243. Naing C, Sitt T, Aung AT, et al. Sustained virologic response to a dual peginterferon alfa-2a and ribavirin in treating chronic hepatitis C infection: a retrospective cohort study. Medicine (Baltimore). 2015;94(30):e1234.

244. Namazee N, Sali S, Asadi S, et al. Real response to therapy in chronic hepatitis C virus patients: a study from iran. Hepat Mon. 2012;12(9):e6151.

245. Nawaz A, Chaudhry A, Riaz M, et al. Treatment of chronic hepatitis C - SVR rates and compliance with conventional interferon therapy and factors influencing response rates. American Journal of Gastroenterology. 2011;106:S125.

246. Nelson D, Bernstein D, Freilich B, et al. All-oral 12-week treatment with daclatasvir and sofosbuvir in treatment-experienced patients infected with HCV genotype 3: A subanalysis of the ALLY-3 phase 3 study. J Viral Hepat. 2015;22:30.

247. Nelson D, Bernstein D, Freilich B, et al. All-oral 12-week combination treatment with daclatasvir (DCV) and sofosbuvir (SOF) in treatment-experienced patients infected with HCV genotype (GT) 3: A subanalysis of the ALLY-3 phase 3 study. Gastroenterology. 2015;148(4):S1003.

248. Nelson D, Cooper J, Lalezari J, et al. All oral 12 week treatment with daclatasvir plus sofosbuvir in patients with chronic hepatitis C virus genotype 3 infection ally 3 phase 3 study. Hepatol Int. 2015;9(1):S56.

249. Nelson DR, Cooper JN, Lalezari JP, et al. All-oral 12-week treatment with daclatasvir plus sofosbuvir in patients with hepatitis C virus genotype 3 infection: ALLY-3 phase III study. Hepatology. 2015;61(4):1127–35.

250. Nelson DR, Feld J, Kowdley KV, et al. All oral therapy with sofosbuvir + ribavirin for 12 or 16 weeks in treatment experienced GT2/3 HCV-infected patients: Results of the phase 3 fusion trial. J Hepatol. 2013;58:S3–S4.

251. Neukam K, Caruz A, Rivero-Juárez A, et al. Genetic variations in proprotein convertase subtilisin/kexin type 9 (PCSK9) gene are associated with response to pegylated interferon/ribavirin in hepatitis C virus genotype 3-infected patients. J Hepatol. 2013;58:S362.

252. Ng T, Pilot-Matias T, Tripathi R, et al. Analysis of HCV genotype 2 and 3 variants in patients treated with combination therapy of next generation HCV direct-acting antiviral agents ABT-493 and ABT-530. J Hepatol. 2016;64:S409–10.

253. Niederau C, Mauss S, Schober A, et al. Predictive factors for sustained virological response after treatment with pegylated interferon alpha-2a and ribavirin in patients infected with HCV genotypes 2 and 3. PLoS One. 2014;9(9):e107592.

254. Nimer A, Mouch A. Vitamin D improves viral response in hepatitis C genotype 2-3 naive patients. World J Gastroenterol. 2012;18(8):800–5.

255. Nyberg LM, Lalezari J, Ni L, et al. Successful retreatment with sofosbuvir-containing regimens for HCV genotype 2 or 3 infected patients who failed prior sofosbuvir plus ribavirin therapy. Gastroenterology. 2014;146(5):S–905.

256. Odolini S, Amadasi S, Cerini C, et al. Sustained virological response to peginterferon therapy in patients infected with HCV (genotypes 2 and 3), with or without HIV. BMC Infect Dis. 2014;14(Suppl 5):S4.

257. Ouzan D, Fontanges T, Pénaranda G, et al. Safety and efficacy of sofosbuvir containing regimens for hepatitis C: Community treatment of a real world population with advanced liver fibrosis. J Hepatol. 2015;62:S645.

258. Ouzan D, Larrey D, Habersetzer F, et al. Virological response and relapse rates in French CHC patients treated with peginterferon alfa-2A/ribavirin: A sub-analysis of the final population from the PROPHESYS study. J Hepatol. 2012;56:S453.

259. Pandya P, Rzouq F, Oni O. Sustained virologic response and other potential genotype-specific roles of statins among patients with hepatitis C-related chronic liver diseases. Clin Res Hepatol Gastroenterol. 2015;39(5):555–65.

260. Patel K, Gordon SC, Sheikh AM, et al. Efficacy and safety of sofosbuvir in patients according to fibrosis stage: An analysis of phase 3 data. Hepatology. 2013;58(4):738A–9A.

261. Pavlidis C, Panoutsopoulos GI, Tiniakos D, et al. Serum leptin and ghrelin in chronic hepatitis C patients with steatosis. World J Gastroenterol. 2011;17(46):5097–104.

262. Pawlotsky JM, Flisiak R, Sarin SK, et al. Alisporivir plus ribavirin, interferon free or in combination with pegylated interferon, for hepatitis C virus genotype 2 or 3 infection. Hepatology. 2015;62(4):1013–23.

263. Pawlotsky JM, Sarin SK, Foster G, et al. Alisporivir plus ribavirin is highly effective as interferon-free or interferon-add-on regimen in previously untreated HCV-GT2 or GT3 patients: SVR12 results from VITAL-1 Phase 2B study. J Hepatol. 2012;56:S553.

264. Pedersen C, Alsio A, Lagging M, et al. Ribavirin plasma concentration is a predictor of sustained virological response in patients treated for chronic hepatitis C virus genotype 2/3 infection. J Viral Hepat. 2011;18(4):245–51.

265. Persico M, Coppola N, Rosato V, et al. HCV antiviral therapy in injection drug users: difficult to treat or easy to cure? Ann Hepatol. 2015;14(3):325–32.

266. Petersen J, Welzel T, Herzer K, et al. Daclatasvir plus sofosbuvir with or without ribavirin for the treatment of chronic HCV infection in patients with decompensated cirrhosis: results of a European multicentre compassionate use program. J Hepatol. 2016;64:S781–2.

267. Pianko S, Cooper C, Brown A, et al. Sofosbuvir + peginterferon/ribavirin for 12 weeks vs. Sofosbuvir + Ribavirin for 16 or 24 weeks in genotype 3 HCV infected patients and treatment-experienced cirrhotic patients with genotype 2 HCV: The BOSON study. J Gastroenterol Hepatol (Australia). 2015;30:90.

268. Pianko S, Flamm SL, Shiffman ML, et al. Sofosbuvir plus velpatasvir combination therapy for treatment- Experienced patients with genotype 1 or 3 hepatitis c virus infection. Ann Intern Med. 2015;163(11):809–17.

269. Pianko S, Flamm SL, Shiffman ML, et al. High efficacy of treatment with sofosbuvir+GS-5816 ±ribavirin for 12 weeks in treatment experienced patients with genotype 1 or 3 HCV infection. Hepatology. 2014;60:297A–8A.

270. Pianko S, Jacobson I, Sulkowski M, et al. Treatment with sofosbuvir + ribavirin for 12 weeks achieves SVR12 of 78% in GT2/3 interferon-ineligible,-intolerant, or-unwilling patients: Tthe POSITRON trial. Hepatol Int. 2013;7:S436.

271. Pianko S, Lawitz E, Poordad F, et al. Sofosbuvir in combination with PEG IFN and ribavirin for 12 weeks provides high SVR rates in HCV-infected genotype 2 or 3 treatment experienced patients with and without compensated cirrhosis: Results from the LONESTAR-2 study. J Gastroenterol Hepatol (Australia). 2014;29:94.

272. Pianko S, Nelson DR, Gordon SC, et al. Sofosbuvir and ribavirin achieves high SVR in patients with genotype 2 or 3 HCV infection who are without treatment options. J Gastroenterol Hepatol. 2013;28:164–5.

273. Pianko S, Zeuzem S, Chuang WL, et al. Randomized trial of albinterferon alfa-2b every 4 weeks for chronic hepatitis C virus genotype 2/3. J Viral Hepat. 2012;19(9):623–34.

274. Pineda JA, Caruz A, Di Lello FA, et al. Low-density lipoprotein receptor genotyping enhances the predictive value of IL28B genotype in HIV/hepatitis C virus-coinfected patients. Aids. 2011;25(11):1415–20.

275. Ponnuswamy SK, Ching Hun T, Kontorinis N, et al. Does baseline ribavirin dose affect sustained virological response in chronic hepatitis C genotype 3 infection? J Gastroenterol Hepatol. 2012;27:172.

276. Ponnuswamy SK, Hun TC, Kontorinis N, et al. The effect of racial differences on hepatitis C treatment response-genotype1 versus genotype 3 infections. J Gastroenterol Hepatol. 2012;27:172.

277. Ponnuswamy SK, Kontorinis N, Kong JY, et al. Predictors of sustained virological response following treatment with pegylated interferon and ribavirin for genotype 3 chronic hepatitis C virus infection in a tertiary centre in Western Australia. J Gastroenterol Hepatol. 2012;27:171.

278. Poordad F, Landis CS, Asatryan A, et al. High antiviral activity of NS5A inhibitor ABT-530 with paritaprevir/ritonavir and ribavirin against hepatitis C virus genotype 3 infection. Liver Int. 2016.

279. Poordad F, Lawitz E, Gutierrez JA, et al. C-SWIFT: Grazoprevir/elbasvir + sofosbuvir in cirrhotic and noncirrhotic, treatment-naive patients with hepatitis C virus genotype 1 infection, for durations of 4, 6 or 8 weeks and genotype 3 infection for durations of 8 or 12 weeks. J Hepatol. 2015;62:S192–3.

280. Poordad F, Schiff ER, Vierling JM, et al. Daclatasvir with sofosbuvir and ribavirin for hepatitis C virus infection with advanced cirrhosis or post-liver transplantation recurrence. Hepatology. 2016;63(5):1493–505.

281. Preveden TA. Steatosis in patients with chronic hepatitis c virus genotype 3 doesn't affect the outcome of peginterferon alpha-2a plus ribavirin treatment. J Hepatol. 2014;60(1):S494.

282. Puoti C, Barbarini G, Picardi A, et al. Rapid virological response as a predictor of sustained response in HCV-infected patients with persistently normal alanine aminotransferase levels: A multicenter study. J Viral Hepat. 2011;18(6):393–9.

283. Qureshi S, Batool U, Iqbal M, et al. Pre-treatment predictors of response for assessing outcomes to standard treatment in infection with HCV genotype 3. J Coll Physicians Surg Pak. 2011;21(2):64–8.

284. Rahman MZ, Perveen S, Mahmuduzzaman M, et al. Peginterferon α-2a and rivabirin in the treatment of chronic hepatitis C. Mymensingh Med J. 2014;23(2):335–40.

285. Rallon NI, Soriano V, Naggie S, et al. Impact of IL28B gene polymorphisms on interferon-lambda3 plasma levels during pegylated interferon-alpha/ribavirin therapy for chronic hepatitis C in patients coinfected with HIV. J Antimicrob Chemother. 2012;67(5):1246–9.

286. Rao P, Koshy A, Philip J, et al. Pegylated interferon alfa-2b plus ribavirin for treatment of chronic hepatitis C. World J Hepatol. 2014;6(7):520–6.

287. Raza S, Abbas Z, Jafri W, et al. Interferon-gamma versus amantadine in combination with interferon-alfa-2b and Ribavirin in chronic hepatitis C genotype 3 non-responders and relapsers. J Int Assoc Physicians AIDS Care. 2011;10(3):207.

288. Reddy KR, Stedman C, Workowski K, et al. An integrated safety and efficacy analysis of sofosbuvir based regimens in patients with hereditary bleeding disorders. J Hepatol. 2016;64:S783.

289. Reddy KR, Workowski K, Terrault N, et al. Sofosbuvir regimens are safe and highly effective in patients with hereditary bleeding disorders. Hepatol Int. 2016;10(1):S16.

290. Rehan HS, Manak S, Yadav M. Supervised conventional interferon α2a in combination with ribavirin therapy is the preferred alternative for treatment of chronic hepatitis C. Indian J Pharmacol. 2014;46(5):490–2.

291. Rembeck K, Waldenstrom J, Hellstrand K, et al. Variants of the inosine triphosphate pyrophosphatase gene are associated with reduced relapse risk following treatment for HCV genotype 2/3. Hepatology. 2014;59(6):2131–9.

292. Restivo L, Zampino R, Guerrera B, et al. Steatosis is the predictor of relapse in HCV genotype 3- but not 2-infected patients treated with 12 weeks of pegylated interferon-α-2a plus ribavirin and RVR. J Viral Hepat. 2012;19(5):346–52.

293. Rivero-Juarez A, Lopez-Cortes LF, Camacho A, et al. A 24-week treatment strategy with pegylated interferon/ribavirin in HIV/hepatitis C virus genotype 3-coinfected patients who achieved a rapid virologic response results in a high sustained virologic response rate. Clin Infect Dis. 2014;58(1):130–3.

294. Roberts SK, Mangia A, Pianko S, et al. Sofosbuvir/velpatasvir for 12 wks vs sofosbuvir + ribavirin for 24 wks in GT3 HCV: The ASTRAL-3 study. Hepatol Int. 2016;10(1):S11–2.

295. Rockstroh JK, Puoti M, Rodriguez-Torres M, et al. Sofosbuvir and ribavirin therapy for the treatment of HIV/HCV coinfected patients with HCV GT1-4 infection: The photon-1 and-2 trials. Hepatology. 2014;60:295A–6A.

296. Rockstroh JK, Welzel TM, Ingiliz P, et al. Daclatasvir plus sofosbuvir with or without ribavirin for the treatment of chronic HCV in patients coinfected with HIV: Interim results of a multicenter compassionate use program. Hepatology. 2015;62:728A–9A.

297. Rodriguez-Torres M, Gaggar A, Shen G, et al. Sofosbuvir for chronic hepatitis C virus infection genotype 1-4 in patients coinfected with HIV. J Acquir Immune Defic Syndr. 2015;68(5):543–9.

298. Roeder C, Jordan S, Schulze Zur Wiesch J, et al. Age-related differences in response to peginterferon alfa-2a/ribavirin in patients with chronic hepatitis C infection. World J Gastroenterol. 2014;20(31):10984–93.

299. Rosina F, Maria Elena T, Borghesio E, et al. PEG-IFN for chronic hepatitis C in clinical practice: The prospective phase of the AIFA study. Digestive and Liver Disease. 2012;44:S101.

300. Rosina F, Tosti ME, Borghesio E, et al. Pegylated interferon alpha plus ribavirin for the treatment of chronic hepatitis C: a multicentre independent study supported by the Italian Drug Agency. Dig Liver Dis. 2014;46(9):826–32.

301. Rosina F, Tosti ME, Borghesio E, et al. PEG-IFN for chronic hepatitis C in clinical practice: The prospective phase of the AIFA study. J Hepatol. 2012;56:S457.

302. Rosina F, Tosti ME, Borghesio E, et al. PEG-IFN for hepatitis C in clinical practice: Results of the retrospective phase of the AIFA study. Dig Liver Dis. 2011;43:S89.

303. Sadeghi F, Bokharaei-Salim F, Salehi-Vaziri M, et al. Associations between human TRIM22 gene expression and the response to combination therapy with Peg-IFNα-2a and ribavirin in Iranian patients with chronic hepatitis C. J Med Virol. 2014;86(9):1499–506.

304. Saigal S, Choudhary NS, Saraf N, et al. Genotype 3 and higher low-density lipoprotein levels are predictors of good response to treatment of recurrent hepatitis C following living donor liver transplantation. Indian J Gastroenterol. 2015;34(4):305–9.

305. Saigal S, Choudhary NS, Saraf N, et al. Genotype 3 and higher LDL levels are predictors of good response for hepatitis C recurrence post living donor liver transplantation. Liver Transpl. 2014;20:S245.

306. Saigal S, Saraf N, Kakodkar R, et al. Pegylated interferon and ribavirin combination is effective in inducing sustained viral response (SVR) in non-genotype 1 patients with post transplant hepatitis C recurrence following living donor liver transplantation. Liver Transpl. 2012;18:S222-3.

307. Santana J, Rockstroh J, Puoti M, et al. Sofosbuvir and ribavirin therapy for the treatment of HIV/HCV co-infected patients with HCV GT1-4 infection: The PHOTON-1 and -2 Trials. J Int AIDS Soc. 2015;18:6–7.

308. Saraswat V, Morakhia N, Pandey G, et al. Impact of support with erythropoietin and granulocyte colony stimulating factor on outcome of antiviral therapy in treatment naïve chronic hepatitis C patients from northern India. Hepatol Int. 2015;9(1):S276.

309. Selic Kurincic T, Lesnicar G, Poljak M, et al. Impact of added fluvastatin to standard-of-care treatment on sustained virological response in Naïve chronic hepatitis C patients infected with genotypes 1 and 3. Intervirology. 2014;57(1):23–30.

310. Selic Kurincic T, Lesnicar G, Poljak M, et al. Addition of fluvastatin to standard of care treatment does not improve sustained virological response in chronic hepatitis c genotype 3 patients. J Hepatol. 2013;58:S357.

311. Shafran S, Shaw D, Cohen E, et al. high rates of SVR in patients with HCV genotype 2 or 3 infection treated with ombitasvir/paritaprevir/r and sofosbuvir with or without ribavirin. Hepatology. 2015:LB-16.

312. Shah S, Acharya SK, Mehta R, et al. Sofosbuvir plus ribavirin in the treatment of chronic HCV infection in India. Hepatol Int. 2016;10(1):S15.

313. Sharma RK, Sikri T, Nayyar SB, et al. Clinical efficacy of pegylated interferon and ribavirin combination therepy in chronic hepatitis C patients. JIACM. 2015;16(2):118–24.

314. Shiffman ML, Cheinquer H, Berg CP, et al. Extended treatment with pegylated interferon alfa/ribavirin in patients with genotype 2/3 chronic hepatitis C who do not achieve a rapid virological response: final analysis of the randomised N-CORE trial. Hepatol Int. 2014;8(4):517–26.

315. Shiffman ML, Parruti G, Ferenci P, et al. Baseline viral load thresholds predicting virological response to peginterferon alfa-2a/ribavirin in HCV G2 and 3 patients: PROPHESYS cohort sub-analysis. Hepatol Int. 2013;7:S356–7.

316. Shoeb D, Dearden J, Weatherall A, et al. Extended duration therapy with pegylated interferon and ribavirin for patients with genotype 3 hepatitis C and advanced fibrosis: Final results from the STEPS trial. J Hepatol. 2014;60(4):699–705.

317. Shoeb D, Rowe IA, Freshwater D, et al. Response to antiviral therapy in patients with genotype 3 chronic hepatitis C: fibrosis but not race encourages relapse. Eur J Gastroenterol Hepatol. 2011;23(9):747–53.

318. Silva GF, Villela-Nogueira CA, Mello CE, et al. Peginterferon plus ribavirin and sustained virological response rate in HCV-related advanced fibrosis: a real life study. Braz J Infect Dis. 2014;18(1):48–52.

319. Sood A, Midha V, Goyal O. Optimal duration of pegylated interferon plus ribavirin therapy for chronic hepatitis C genotype 3 patients who do not achieve rapid virological response. J Clin Exp Hepatol. 2015;5(1):2–7.

320. Sood A, Midha V, Goyal O, et al. Treatment of chronic hepatitis C with pegylated interferon plus ribavirin in treatment-naive 'real-life' patients in India. Hepatol Int. 2014;8(1):S216.

321. Sood A, Midha V, Goyal O, et al. Treatment of chronic hepatitis C with pegylated interferon plus ribavirin in treatment-naive 'real-life' patients in India. Indian J Gastroenterol. 2014;33(4):343–9.

322. Spinzi G, Andreoletti M, Borroni G, et al. Impact of age and sex on response to therapywith pegylated interferon plus ribavirin in genotype 2 and 3 chronic hepatitis C patients. Dig Liver Dis. 2013;45:S212.

323. Stahmeyer JT, Krauth C, Bert F, et al. Costs and outcomes of treating chronic hepatitis C patients in routine care - results from a nationwide multicenter trial. J Viral Hepat. 2016;23(2):105–15.

324. Stattermayer AF, Strassl R, Maieron A, et al. Polymorphisms of interferon-lambda4 and IL28B - effects on treatment response to interferon/ribavirin in patients with chronic hepatitis C. Aliment Pharmacol Ther. 2014;39(1):104–11.

325. Steinebrunner N, Sprinzl MF, Zimmermann T, et al. Early virological response may predict treatment response in sofosbuvir-based combination therapy of chronic hepatitis c in a multi-center "real-life" cohort. BMC Gastroenterol. 2015;15:97.

326. Steinebrunner N, Sprinzl MF, Zimmermann T, et al. Early virological response is a predictor of treatment response in sofosbuvir-based combination therapy of chronic hepatitis c in a multi-center “real-life” cohort. J Viral Hepat. 2015;22:93.

327. Sulkowski MS, Gardiner DF, Rodriguez-Torres M, et al. Daclatasvir plus sofosbuvir for previously treated or untreated chronic HCV infection. N Engl J Med. 2014;370(3):211–21.

328. Sulkowski MS, Naggie S, Lalezari J, et al. Sofosbuvir and ribavirin for hepatitis C in patients with HIV coinfection. JAMA. 2014;312(4):353–61.

329. Tan SS, Leong CL. HIVHCV co-infected patients assessed for treatment in Malaysia-their clinical profiles and response to pegylated interferon and ribavirin. Hepatol Int. 2013;7:S394.

330. Tan SS, Leong CL, Lee CK. Treating hepatitis C in HIV/HCV co-infected patients in Malaysia - the outcomes and challenges. Med J Malaysia. 2015;70(5):281–7.

331. Taneja S, Tohra S, Dhiman RK, et al. Can transient elastography replace liver biopsy for prediction of treatment response with pegylated interferon and ribavarin in chronic hepatitis C?-An experience from a tertiary care hospital. J Gastroenterol Hepatol (Australia). 2015;30:185.

332. Tangkijvanich P, Komolmit P, Mahachai V, et al. Response-guided therapy for patients with hepatitis C virus genotype 6 infection: a pilot study. J Viral Hepat. 2012;19(6):423–30.

333. Tangkijvanich P, Komolmit P, Mahachai V, et al. Individualized treatment according to rapid virologic response in hepatitis C virus genotype 6: Infected patients. Hepatol Int. 2011;5(1):169–70.

334. Tanwandee T, Pithukpakorn M, Vipatakul N, et al. Genetic polymorphism of low-density lipoprotein receptor did not affect treatment outcome of chronic hepatitis C genotype 3. J Med Assoc Thai. 2011;94(Suppl 1):S147–53.

335. Téllez M, Vispo E, Aguirrebengoa K, et al. HCV genotype 3 and subtype 1b, but not 1a, are independent predictors of sustained virological response to pegIFN-RBV in HCV/HIVcoinfected patients with unfavourable IL28B variants. Hepatology. 2011;54:824A.

336. Thanak P, Kitiyakara T, Intaraprasong P, et al. Real-world outcomes of hepatitis C treatment in thai patients. J Gastroenterol Hepatol. 2012;27:247.

337. Thanapirom K, Suksawatamnuay S, Sukeepaisarnjaroen W, et al. Association between CXCL10 and DPP4 gene polymorphisms and a complementary role for unfavorable IL28B genotype in prediction of treatment response in thai patients with chronic hepatitis C virus infection. PLoS One. 2015;10(9):e0137365.

338. Thornton K, Deming P, Sedillo M, et al. Low sustained virologic response (SVR) rates in genotype (GT) 2 and 3 patients with quantifiable hepatitis C virus (HCV) at week 4 of treatment with sofosbuvir (SOF) containing regimens. J Hepatol. 2016;64:S786.

339. Ting AY, Friedman ND, Green J, et al. Hepatitis C virus treatment in the 'Real world': How well do 'Real' patients respond? J Gastroenterol Hepatol. 2012;27:181–2.

340. Tohra SK, Taneja S, Ghosh S, et al. Prediction of sustained virological response to combination therapy with pegylated interferon alfa and ribavirin in patients with genotype 3 chronic hepatitis C. Dig Dis Sci. 2011;56(8):2449–55.

341. Tontodonati M, Cento V, Polilli E, et al. May some HCV genotype 1 patients still benefit from dual therapy? The role of very early HCV kinetics. New Microbiol. 2015;38(4):491–7.

342. Tran HA, Jones TL, Gibson R, et al. Thyroid disease is a favorable prognostic factor in achieving sustained virologic response in chronic hepatitis C undergoing combination therapy: A nested case control study. BMC Endocr Disord. 2011;11:10.

343. Tran TT, Morgan TR, Thuluvath PJ, et al. Safety and efficacy of treatment with sofosbuvir+ GS-5816±ribavirin for 8 or 12 weeks in treatment naïve patients with genotype 1-6 HCV infection. Hepatology. 2014;60:237A.

344. Trombatt WD, Koerner PH, Craft ZN, et al. Retrospective analysis of the medication utilization and clinical outcomes of patients treated with various regimens for hepatitis C infection. J Pharm Pract. 2017;30(2):154–161.

345. Umar M, Khaar HB, Khan SA, et al. Early predictability of virological response in patients of chronic hepatitis-C with genotype-3, treated with pegylated interferon and ribavirin. J Ayub Med Coll Abbottabad. 2014;26(4):559–63.

346. Vasudevan S, Shalimar, Kalra N, et al. Demographic profile, host, disease and viral predictive factors of response in patients with chronic hepatitis C virus infection at a tertiary care hospital in North India. Indian J Gastroenterol. 2014;33(1):A108–9.

347. Velosa J, Serejo F, Bana T, et al. Chronic hepatitis C treated with peginterferon alfa plus ribavirin in clinical practice. Hepatogastroenterology. 2011;58(109):1260–6.

348. Venugopal S. Use of supportive therapy and sustained virological response rates in chronic hepatitis C. Indian J Gastroenterol. 2012;31(1):A59.

349. Verma A, Negi TS, Choudhuri G. Should Indian patients with liver cirrhosis due to HCV genotype 3 be treated? Hepatol Int. 2011;5(1):272–3.

350. Vermehren J, Susser S, Dietz J, et al. Retreatment of patients who failed DAA-combination therapies: real-world experience from a large hepatitis C resistance database. J Hepatol. 2016;64:S188.

351. Vidal-Castineira JR, Lopez-Vazquez A, Alonso-Arias R, et al. A predictive model of treatment outcome in patients with chronic HCV infection using IL28B and PD-1 genotyping. J Hepatol. 2012;56(6):1230–8.

352. Wade AJ, Macdonald DM, Doyle JS, et al. The cascade of care for an australian community-based hepatitis C treatment service. PLoS One. 2015;10(11):e0142770.

353. Wadhawan M, Gupta S, Goyal N. Results of preemptive treatment of hepatitis c genotype 3 after living related liver transplant (LRLT)-based on RVR, a novel approach. Liver Transplantation. 2014;20:S318–9.

354. Waldenström J, Färkkilä M, Rembeck K, et al. Short interferon and ribavirin treatment for HCV genotype 2 or 3 infection: NORDynamIC trial and real-life experience. Scand J Gastroenterol. 2016;51(3):337–43.

355. Waring JF, Dumas EO, Abel S, et al. Serum miR-122 may serve as a biomarker for response to direct acting antivirals: effect of paritaprevir/R with dasabuvir or ombitasvir on miR-122 in HCV-infected subjects. J Viral Hepat. 2016;23(2):96-104.

356. Wedd J, Ford R, Norvell J, et al. Large single center experience of sofosbuvir and ledipasvir therapy for the treatment of patients with hepatitis C virus. J Hepatol. 2016;64:S780.

357. Weis N, Young J, Hofer H, et al. Effectiveness of daclatasvir based therapy in patients with chronic hepatitis C in Europe: Experience from the named patient program. J Hepatol. 2016;64:S801.

358. Welzel T, Herzer K, Ferenci P, et al. Daclatasvir plus sofosbuvir with or without ribavirin for the treatment of HCV in patients with severe liver disease: Interim results of a compassionate use program. J Viral Hepat. 2015;22:4–5.

359. Welzel TM, Herzer K, Ferenci P, et al. Daclatasvir plus sofosbuvir with or without ribavirin for the treatment of HCV in patients with severe liver disease: Interim results of a multicenter compassionate use program. J Hepatol. 2015;62:S619–20.

360. Welzel TM, Nelson DR, Morelli G, et al. Safety and efficacy of sofosbuvir and ribavirin for the treatment of HCV genotype 2 and 3: Results of the HCV-TARGET Study. Hepatology. 2015;62:727A–8A.

361. Welzel TM, Petersen J, Ferenci P, et al. Safety and efficacy of daclatasvir plus sofosbuvir with or without ribavirin for the treatment of chronic HCV genotype 3 infection: Interim results of a multicenter European compassionate use program. Hepatology. 2015;62:225A–6A.

362. Wiegand SB, Heidrich B, Susser S, et al. Performance and value of IFN-Lambda3 and IFN-Lambda4 genotyping in patients with chronic hepatitis C (CHC) genotype 2/3 in a real world setting. PLoS One. 2015;10(12):e0145622.

363. Win KM, Hlaing NKT, Bwa AH, et al. Treatment chronic hepatitis C Myanmar patients with sofosbuvir, pegylated interferon and ribavirin. Hepatol Int. 2016;10(1):S132.

364. Win LL, James P, Wong DK. Response-guided therapy for hepatitis C genotype 2 and 3 in those with HIV coinfection. Dig Dis Sci. 2014;59(8):1946–9.

365. Wu CJ, Roytman MM, Hong LK, et al. Real-world experience with sofosbuvir-based regimens for chronic hepatitis C, including patients with factors previously associated with inferior treatment response. Hawaii J Med Public Health. 2015;74(9 Suppl 2):3–7.

366. Wyles D, Brau N, Kottilil S, et al. Sofosbuvir/velpatasvir for 12 weeks in patients coinfected with HCV and HIV-1: The ASTRAL-5 Study J Hepatol. 2016;64(S2):S188–9.

367. Wyles D, Ruane P, Sulkowski M, et al. Daclatasvir plus sofosbuvir for treatment of HCV genotypes 1-4 in HIV-HCV coinfection: The ALLY-2 study. J Viral Hepat. 2015;22:31–2.

368. Wyles DL, Ruane P, Sulkowski MS, et al. Daclatasvir plus sofosbuvir for treatment of HCV genotypes 1-4 in HIV-HCV coinfection: The ALLY-2 study. J Hepatol. 2015;62:S263.

369. Wyles DL, Ruane PJ, Sulkowski MS, et al. Daclatasvir plus sofosbuvir for HCV in patients coinfected with HIV-1. N Engl J Med. 2015;373(8):714–25.

370. Yamada K, Ishigami M, Kuzuya T, et al. Associations between responses to interferon therapy and genetic variation in interleukin-28B and the core region of hepatitis C virus genotype 3a. J Med Virol. 2015;87(8):1361–7.

371. Yanny BT, Sahota AK. Efficacy and adverse event profile of sofosbuvir based hepatitis C virus (HCV) treatment regimens in the elderly in a community based setting. Hepatology. 2015;62:760A–1A.

372. Younossi ZM, Stepanova M, Estep M, et al. Dysregulation of distal cholesterol biosynthesis in association with relapse and advanced disease in CHC genotype 2 and 3 treated with sofosbuvir and ribavirin. J Hepatol. 2016;64(1):29–36.

373. Yozviak JL, Penaloza OJ, Friel TJ. A novel approach for a new era: Successful integration of multidisciplinary, hepatitis C care within an established HIV primary care practice. Journal of the International Association of Physicians in AIDS Care. 2011;10(3):204–5.

374. Zaman N, Asad MJ, Raza A, et al. Presence of HCV RNA in peripheral blood mononuclear cells may predict patients response to interferon and ribavirin therapy. Ann Saudi Med. 2014;34(5):401–6.

375. Zeuzem S, Arora S, Bacon B, et al. Peginterferon Lambda-1A (Lambda) compared to peginterferon alfa-2A (alfa) in treatment-naive patients with HCV genotypes (G) 2 or 3: First SVR24 results from emerge phase IIB. J Hepatol. 2012;56:S5–6.

376. Zeuzem S, Dusheiko GM, Salupere R, et al. Sofosbuvir and ribavirin in HCV genotypes 2 and 3. N Engl J Med. 2014;370(21):1993–2001.

377. Zeuzem S, Dusheiko GM, Salupere R, et al. Sofosbuvir + ribavirin for 12 or 24 weeks for patients with HCV genotype 2 or 3: the VALENCE trial. Hepatology. 2013;58(4):733A–4A.

378. Zeuzem S, Flisiak R, Hyland RH, et al. Early viral kinetics do not predict treatment outcome with sofosbuvir + ribavirin for 12 or 24 weeks in HCV genotype 2/3 patients in the valence trial. Gastroenterology. 2014;146(5):S978–9.

379. Zhdanov K, Isakov V, Kersey K, et al. Sofosbuvir plus ribavirin for the treatment of Russian patients with chronic HCV genotype 1 or 3 infection. Hepatol Int. 2015;9(1):S52.
