# Supplementary figures and images for: Effectiveness of current and future regimens for treating genotype 3 hepatitis C virus infection: a large-scale systematic review
Source: BMC Infect Dis. 2017 Nov 16;17:722. doi: 10.1186/s12879-017-2820-z (PMC5691805; doi:10.1186/s12879-017-2820-z)

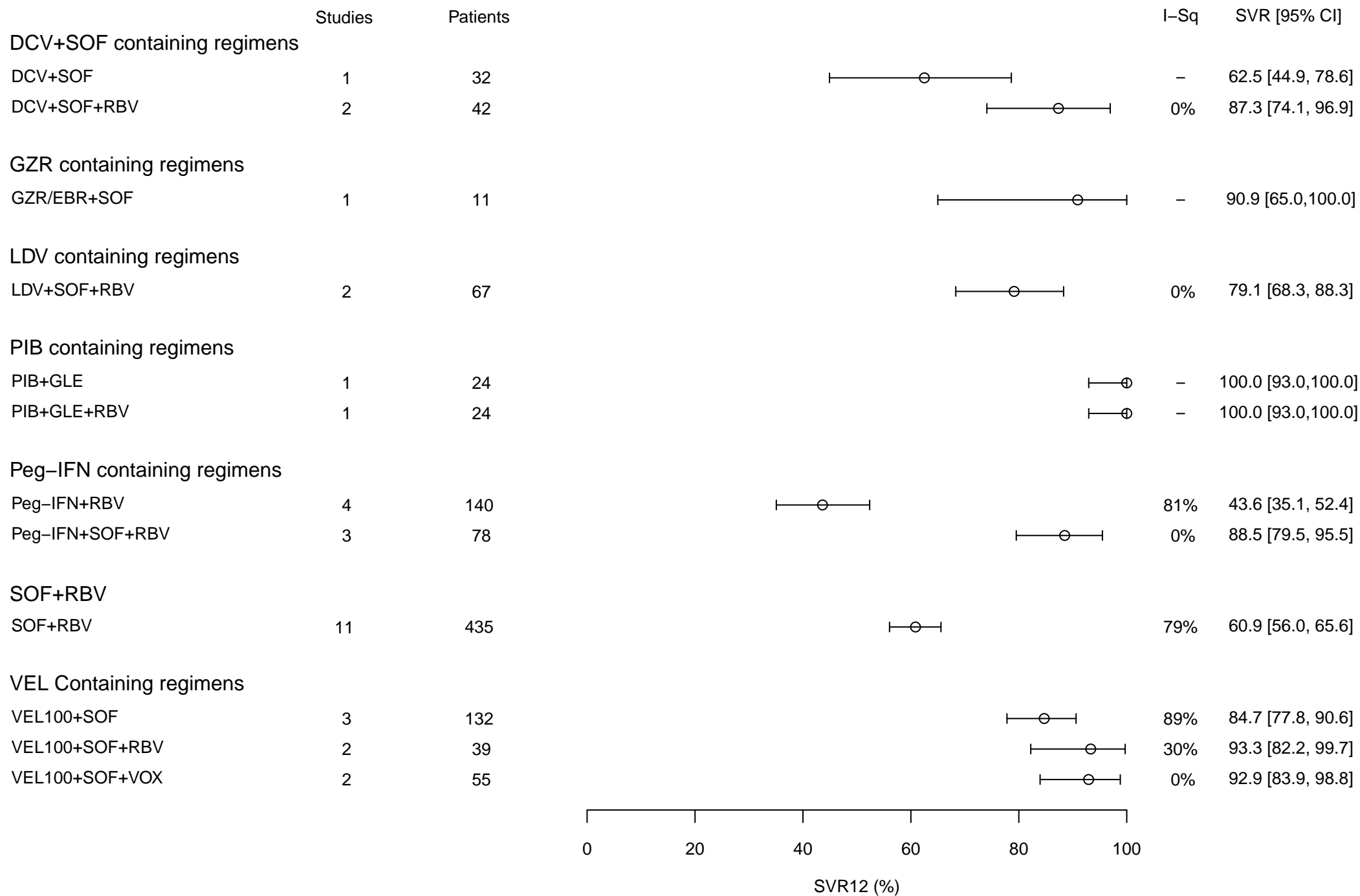

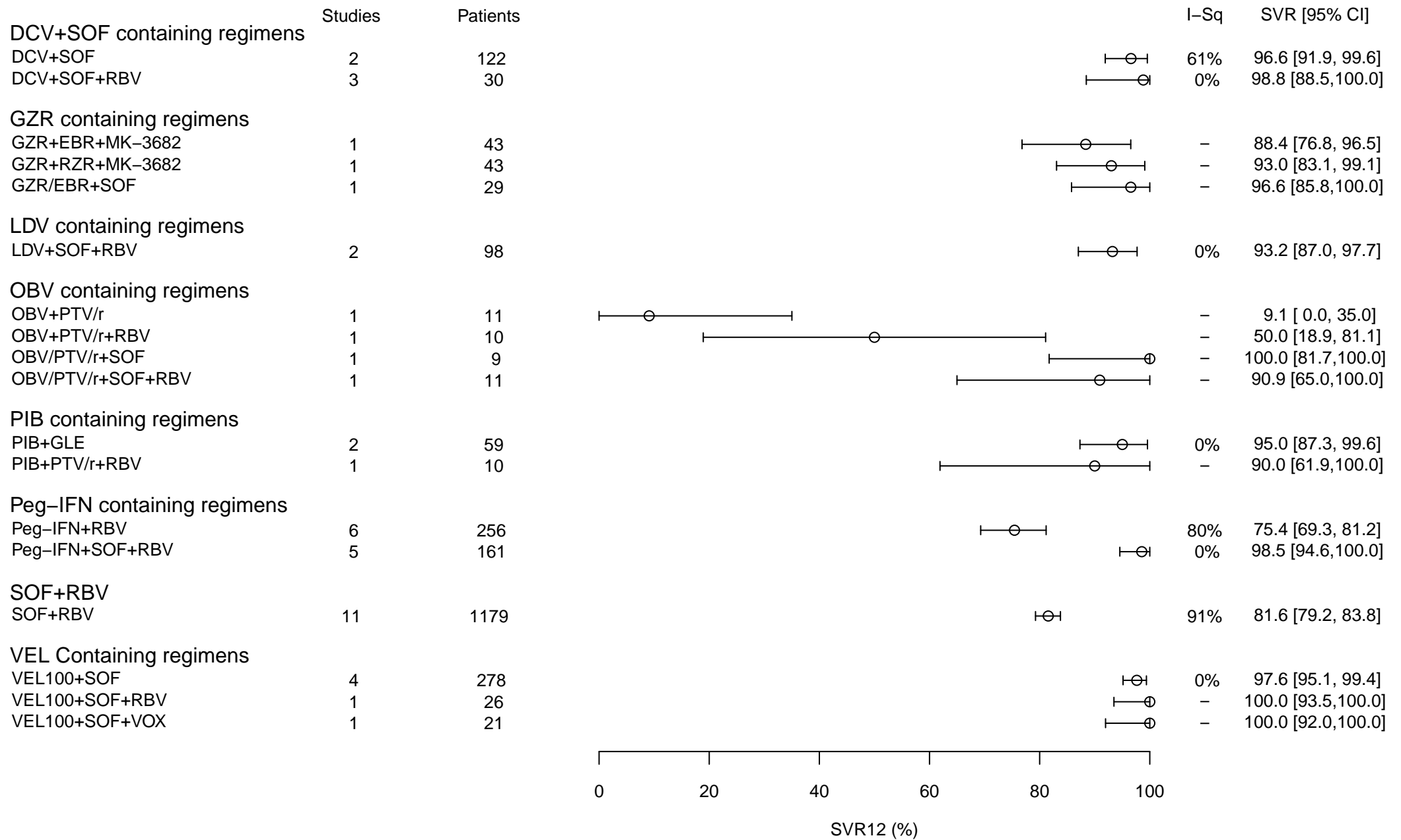

Supplement: Supplementary file 4 — Clinical trial pooled SVR12 rates for (a) patients with cirrhosis and (b) patients without cirrhosis. Forest plot showing SVR12 rates from clinical trials stratified by the presence or absence of cirrhosis. (PDF 11 kb) [file 12879_2017_2820_MOESM4_ESM.pdf]

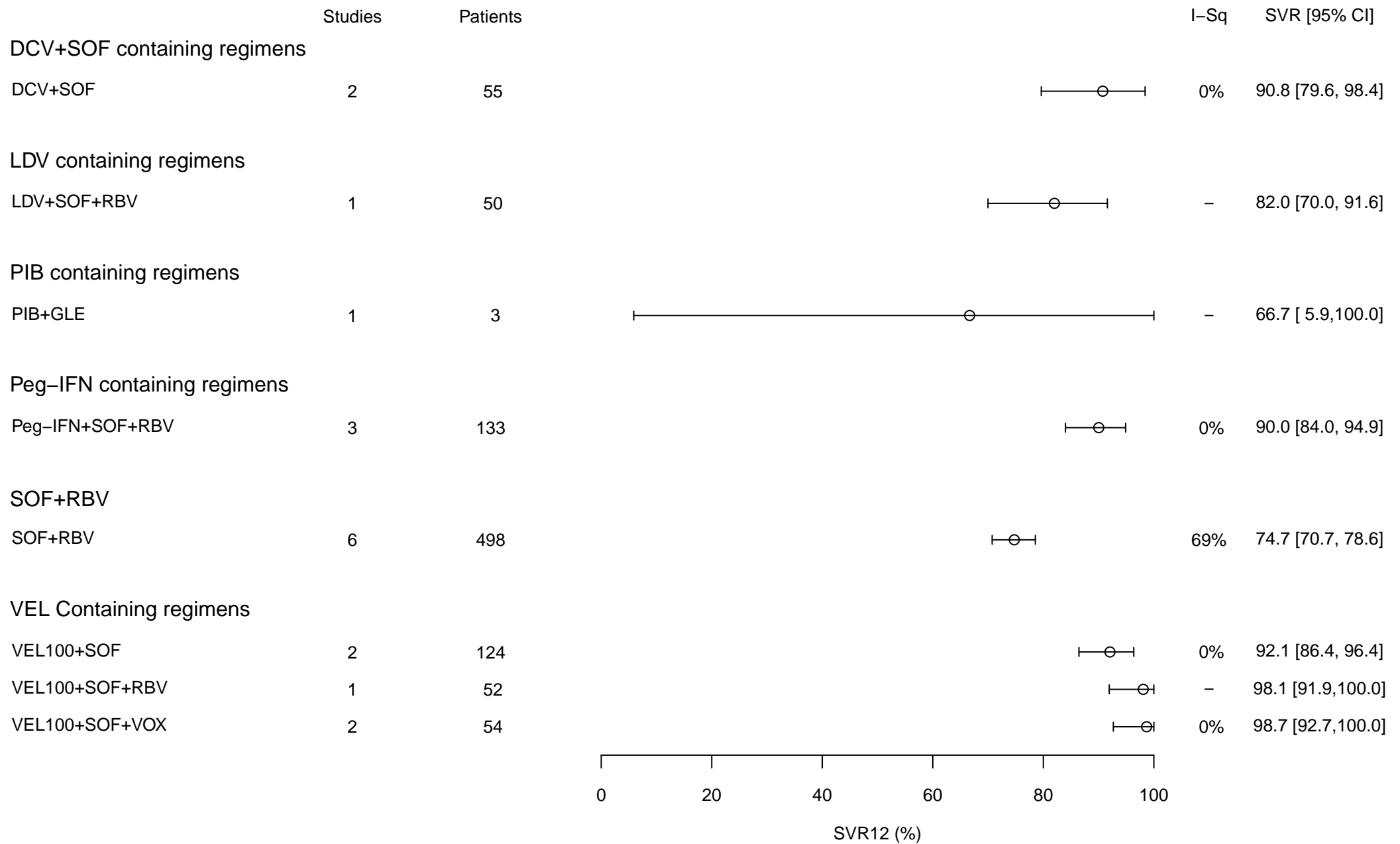

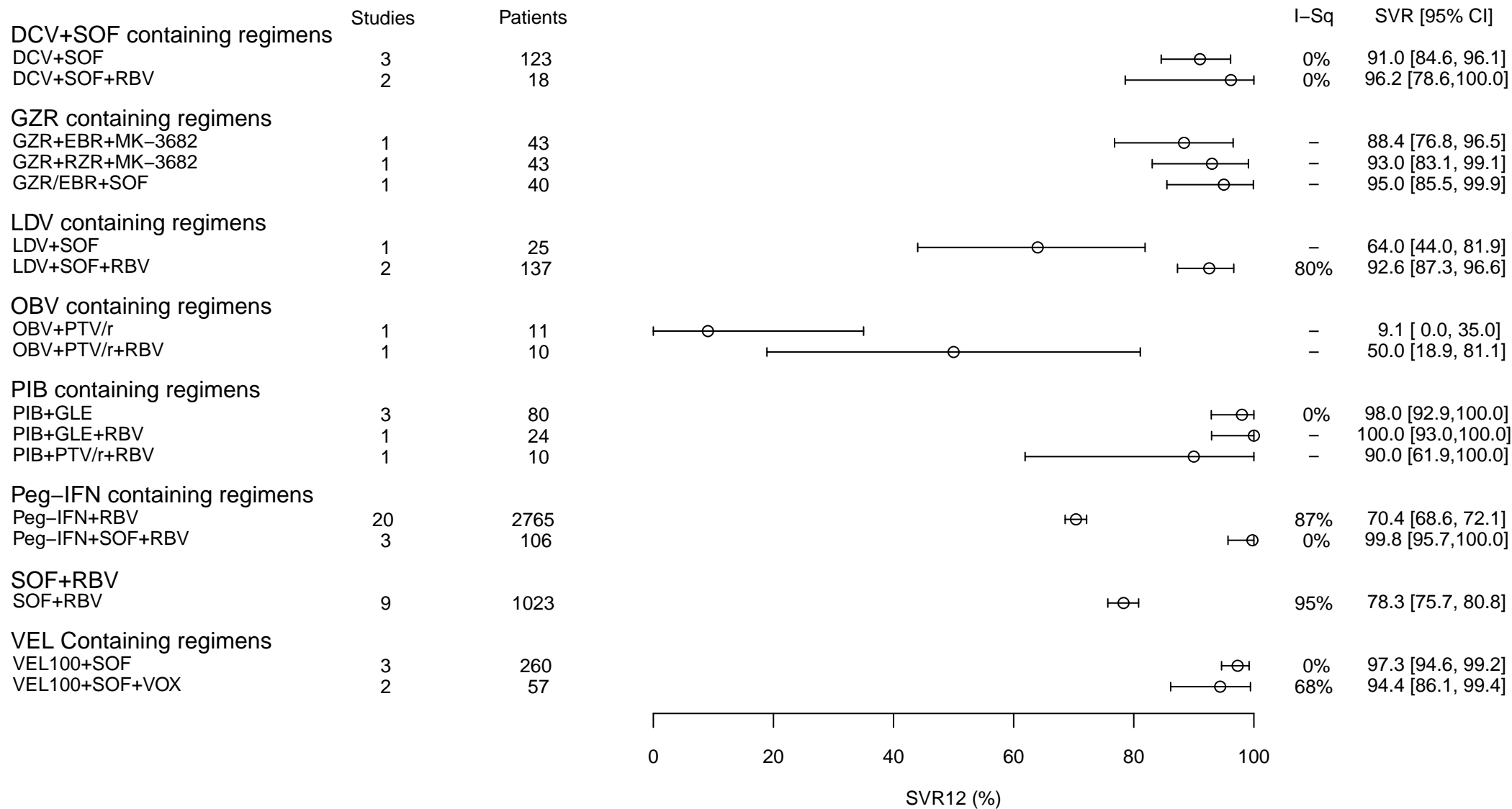

Supplement: Supplementary file 5 — Clinical trial pooled SVR12 rates for (a) treatment-experienced patients and (b) treatment-naïve patients. Forest plot showing SVR12 rates from clinical trials stratified by previous treatment history. (PDF 10 kb) [file 12879_2017_2820_MOESM5_ESM.pdf]

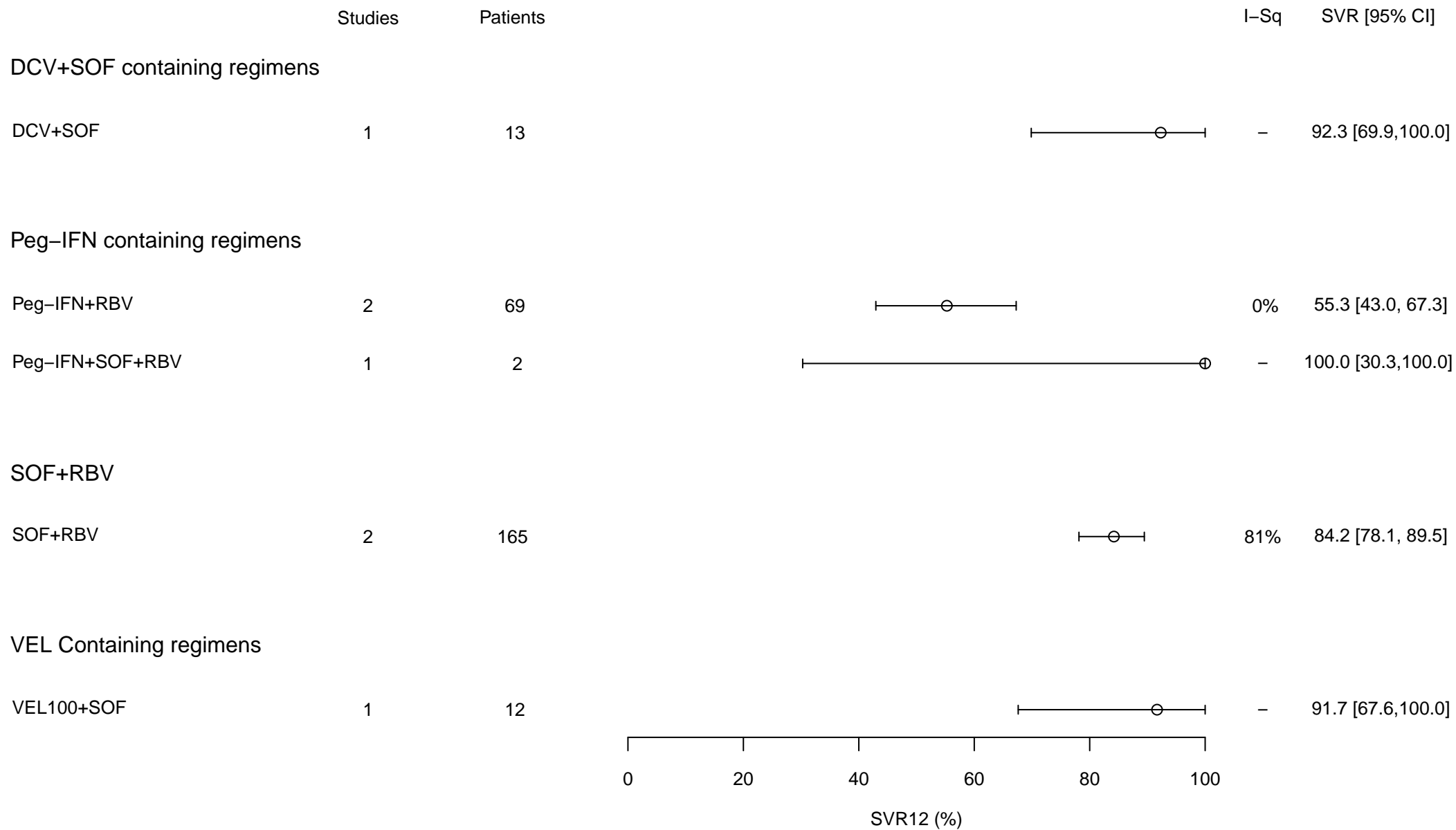

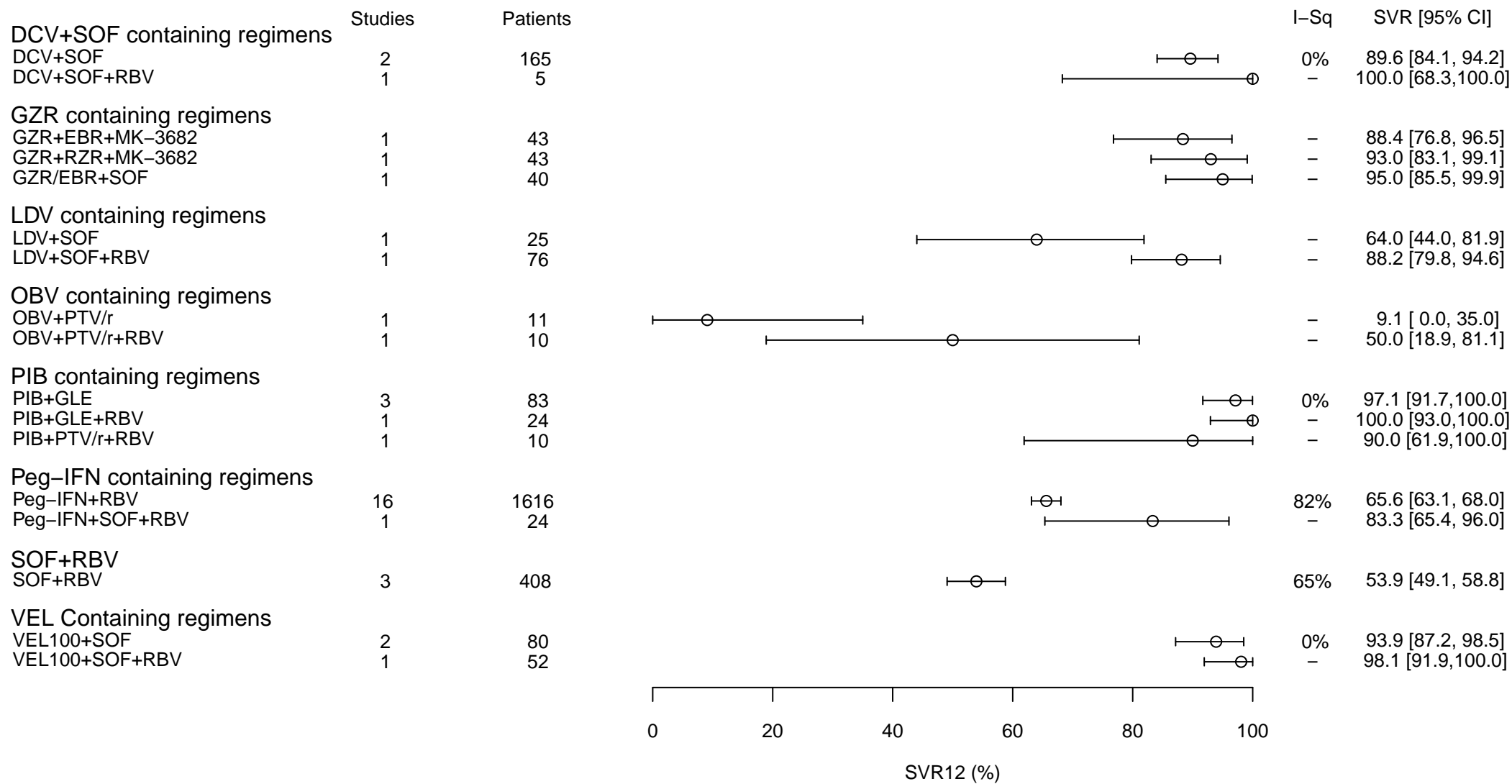

Supplement: Supplementary file 6 — Clinical trial pooled SVR12 rates for (a) patients without HIV and (b) patients with HIV. Forest plot showing SVR12 rates from clinical trials stratified by the presence or absence of HIV. (PDF 10 kb) [file 12879_2017_2820_MOESM6_ESM.pdf]

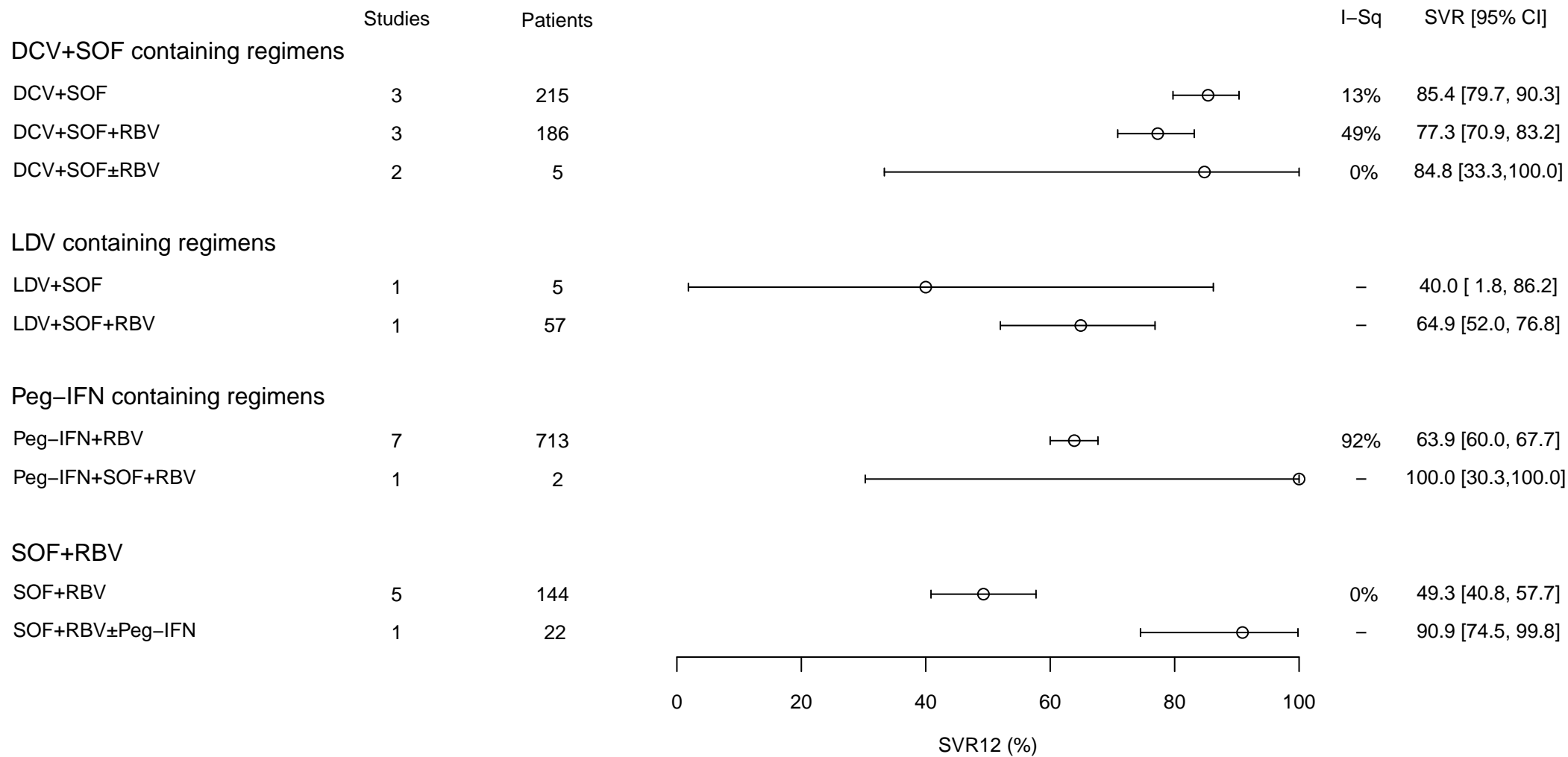

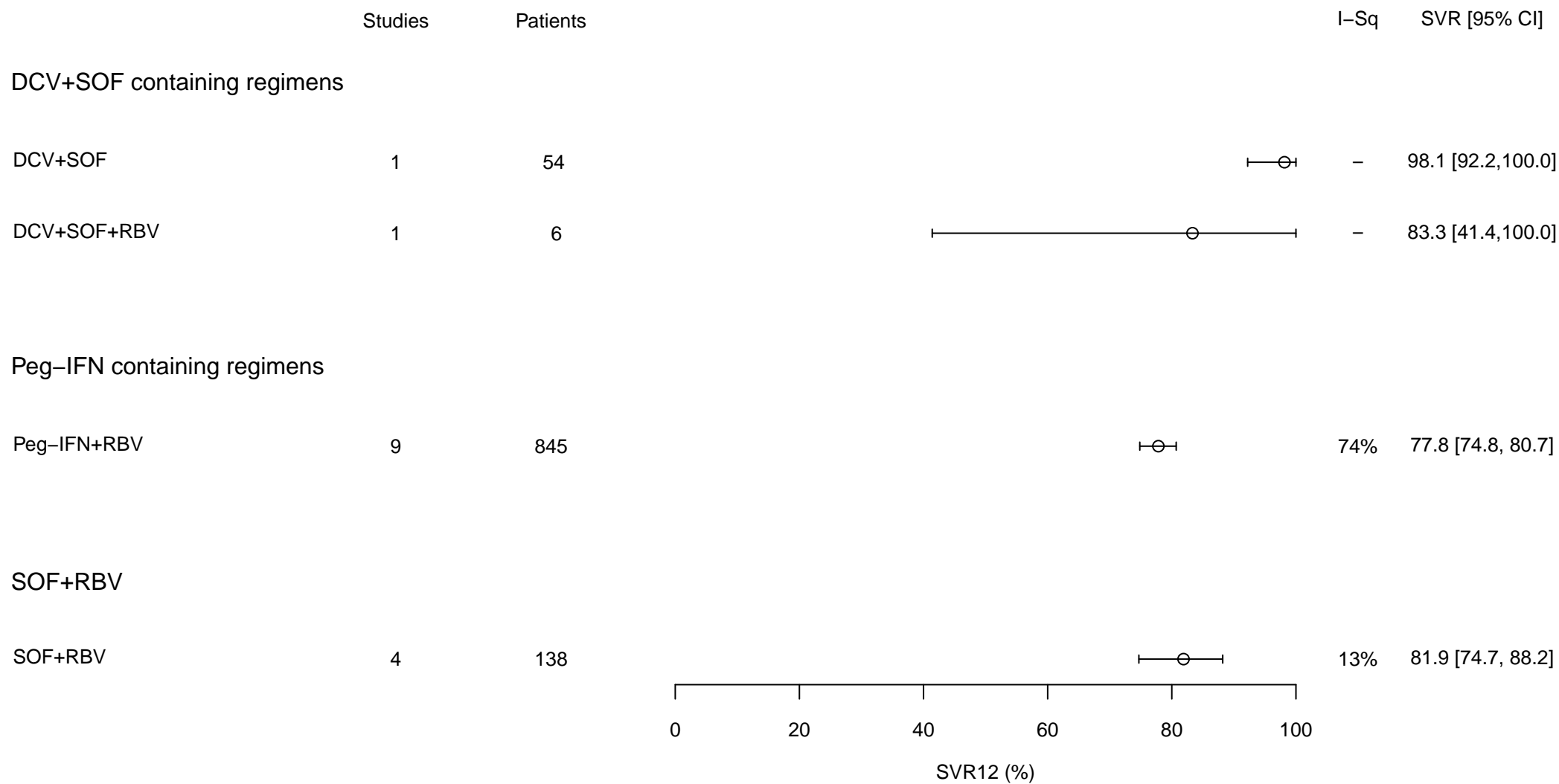

Supplement: Supplementary file 7 — Real-world pooled SVR12 rates for (a) patients with cirrhosis and (b) patients without cirrhosis. Forest plot showing SVR12 rates from real-world datasets stratified by the presence or absence of cirrhosis. (PDF 22 kb) [file 12879_2017_2820_MOESM7_ESM.pdf]

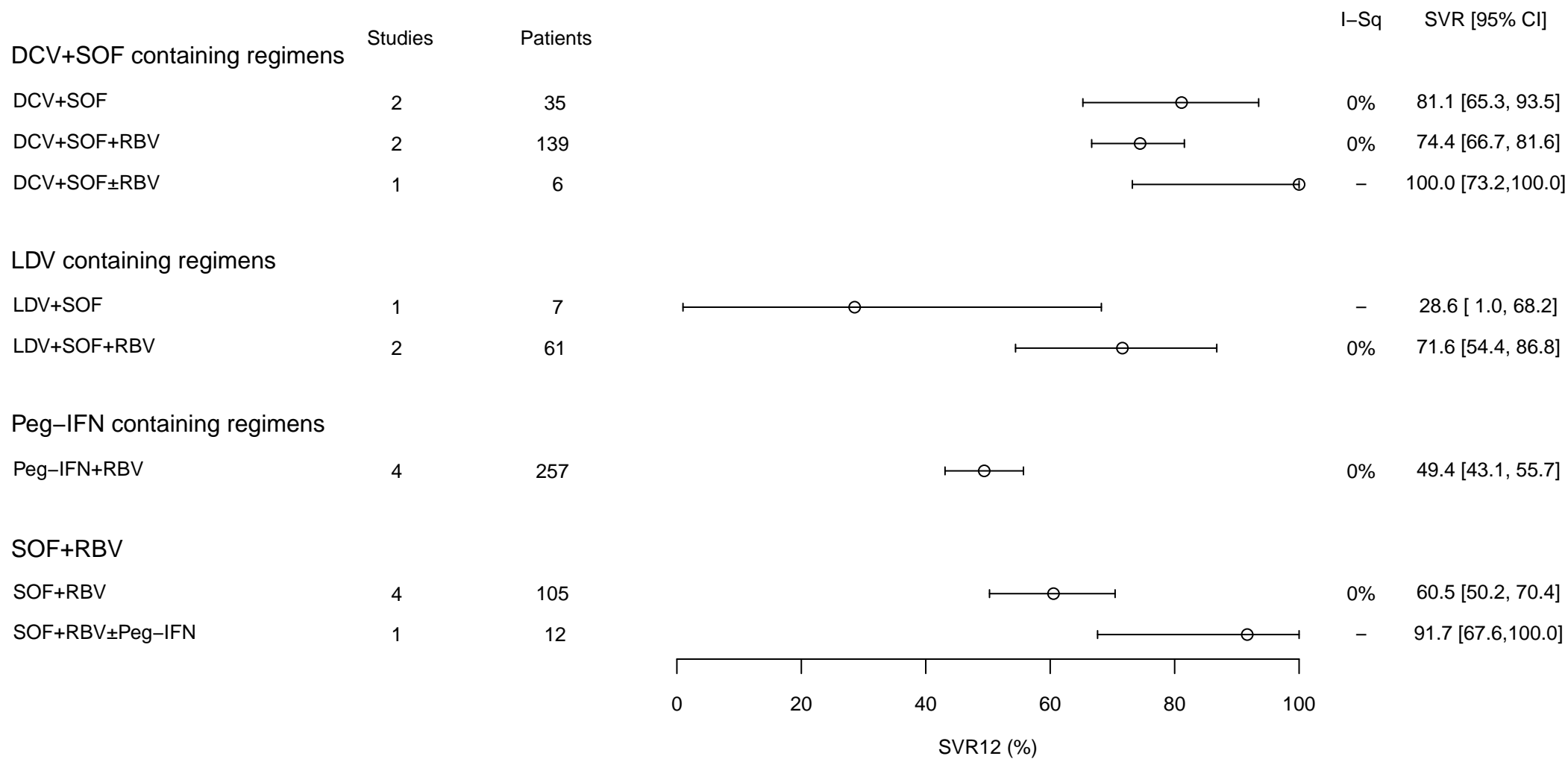

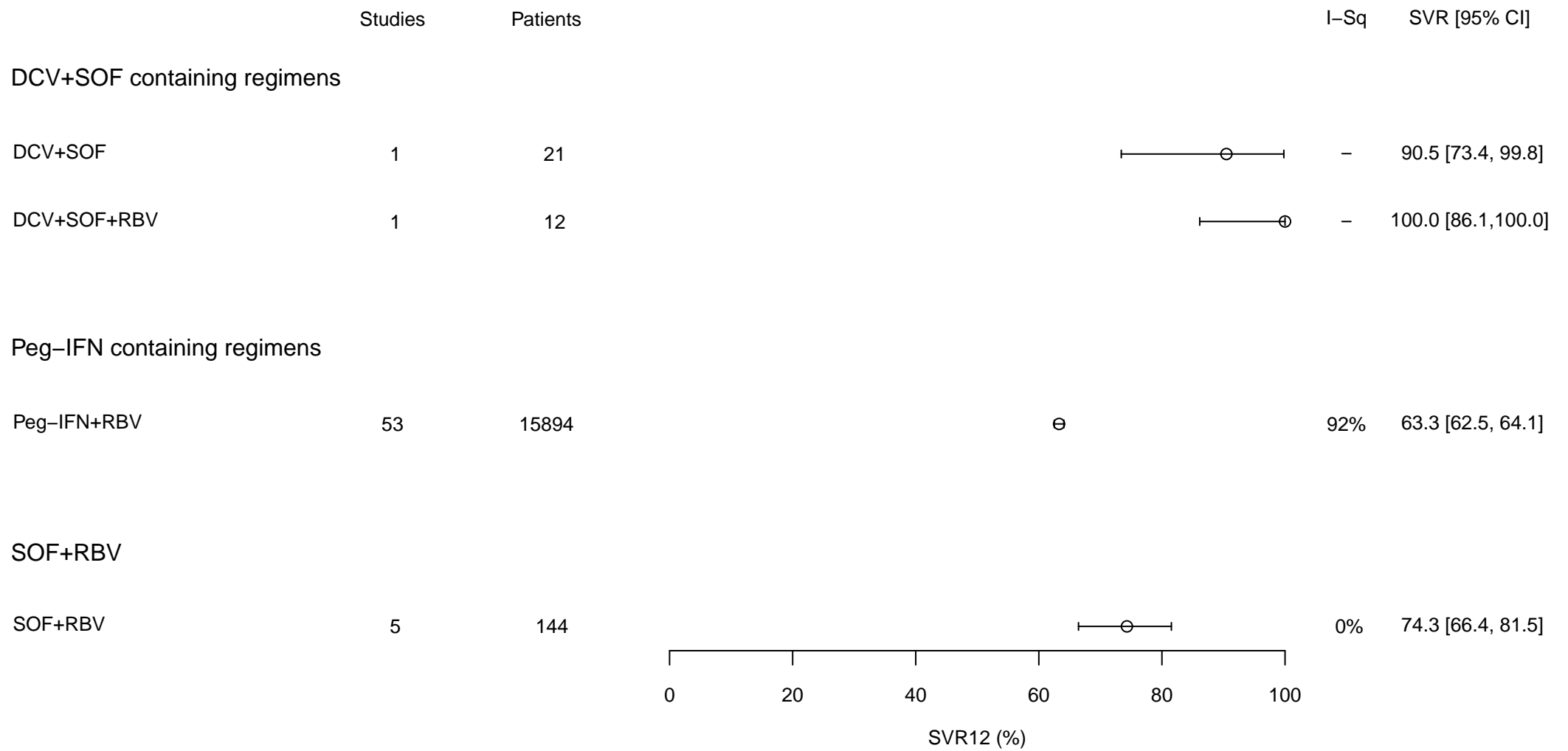

Supplement: Supplementary file 8 — Real world pooled SVR12 rates for (a) treatment-experienced patients and (b) treatment-naïve patients. Forest plot showing SVR12 rates from real-world datasets stratified by previous treatment history. (PDF 31 kb) [file 12879_2017_2820_MOESM8_ESM.pdf]

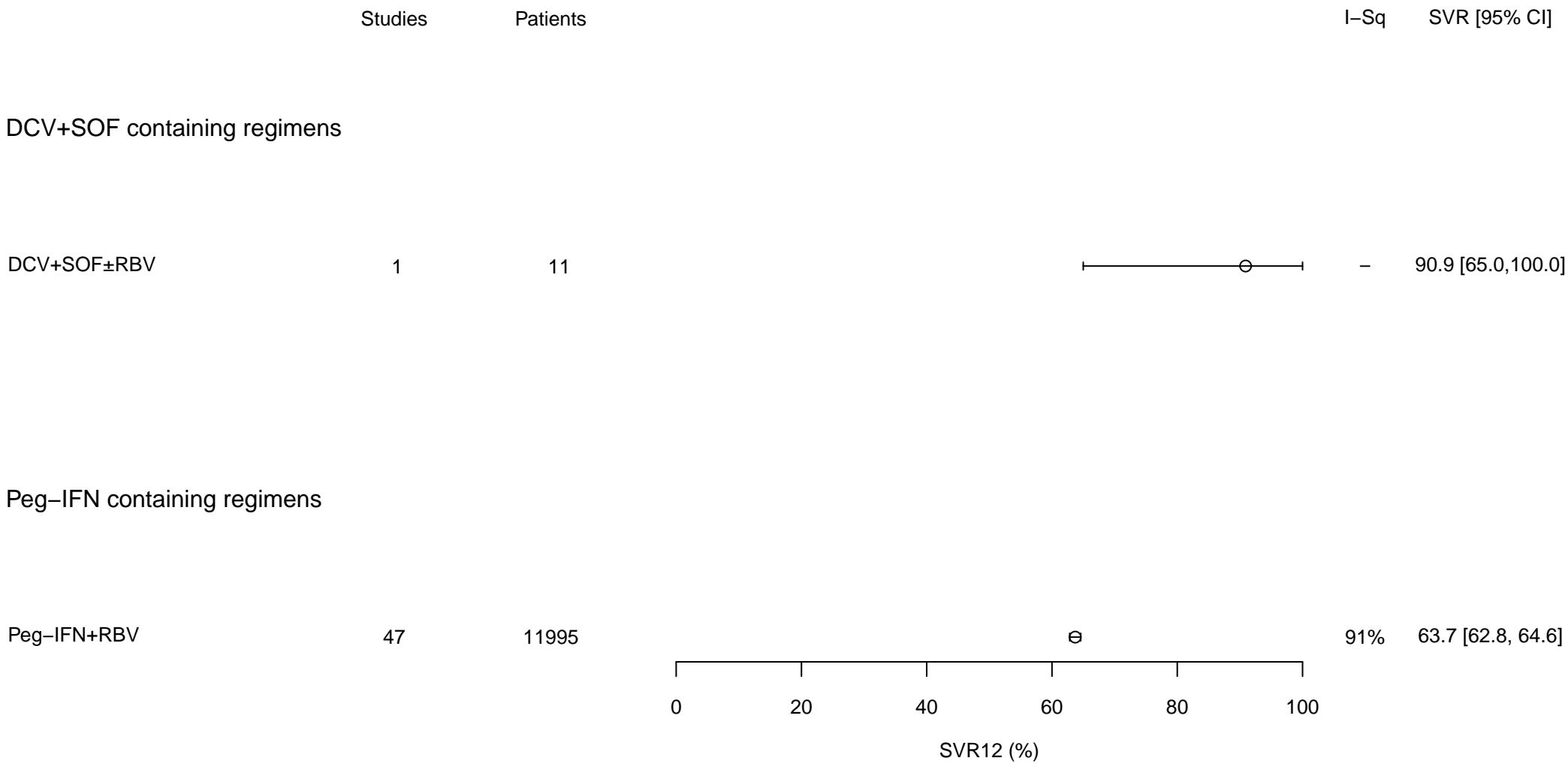

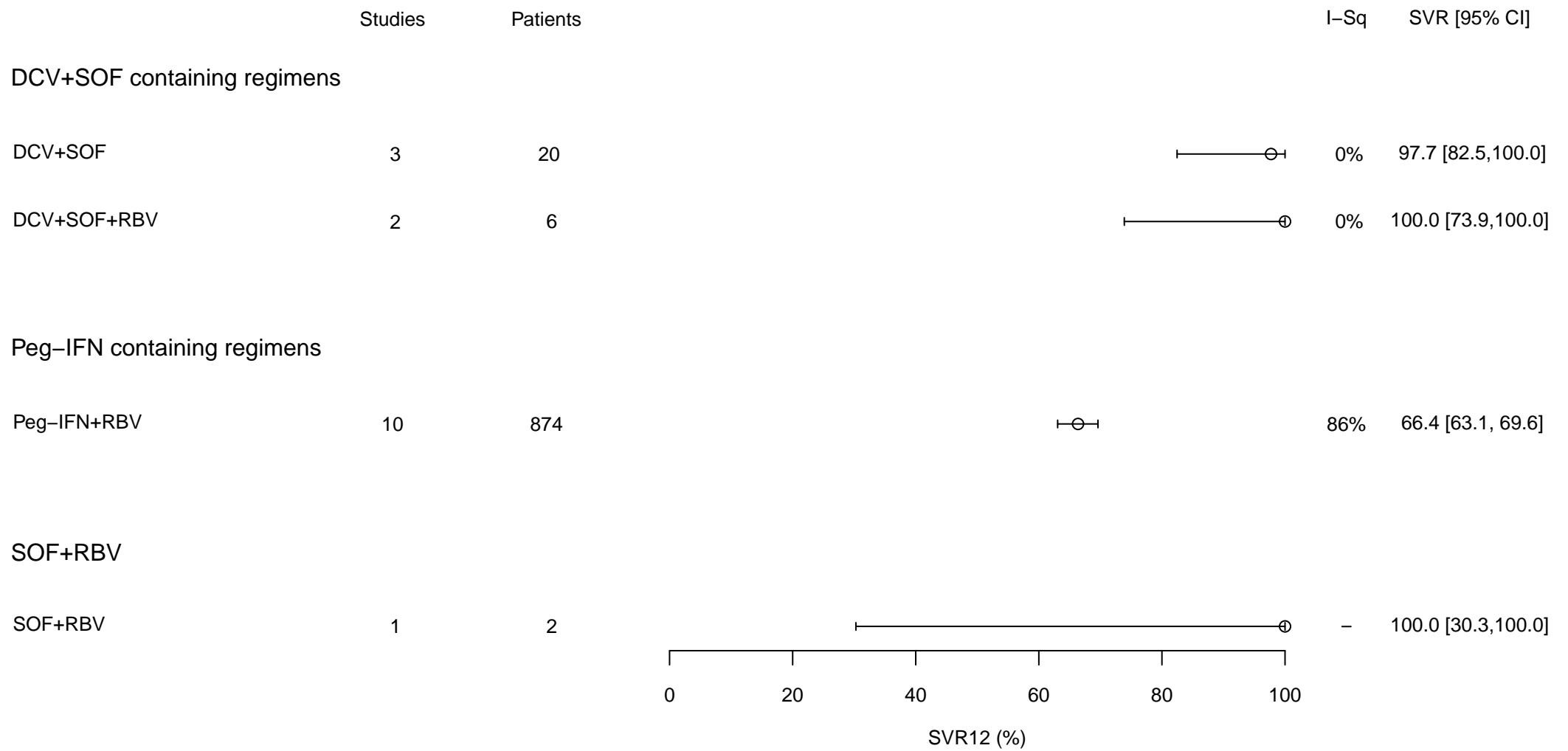

Supplement: Supplementary file 9 — Real world pooled SVR12 rates for (a) patients without HIV and (b) patients with HIV. Forest plot showing SVR12 rates from real-world datasets stratified by the presence or absence of HIV. (PDF 9 kb) [file 12879_2017_2820_MOESM9_ESM.pdf]
